# Supplementary material for: Theoretical Study on the Copper-Catalyzed ortho-Selective C-H Functionalization of Naphthols with α-Phenyl-α-Diazoesters
Source: Molecules. 2023 Feb 13;28(4):1767. doi: 10.3390/molecules28041767 (PMC9960375; doi:10.3390/molecules28041767)
Supplement: Supplementary file 1 [file molecules-28-01767-s001.zip › molecules-2192675-supplementary.pdf]

Supporting Information

Theoretical study on the copper-catalyzed *ortho*-selective C-H functionalization of naphthols with α-phenyl-α-diazoesters

Xiaoli Zhu,[a] Xun-Shen Liu,[a] Fei Xia[a,c,] and Lu Liu[a,b,*]*

a School of Chemistry and Molecular Engineering, East China Normal University, 500 Dongchuan Road Shanghai, 200241, P. R. China

E-mail: lliu@chem.ecnu.edu.cn, *fxia@chem.ecnu.edu.cn*

b Shanghai Engineering Research Center of Molecular Therapeutics and New Drug Development, East China Normal University, Shanghai 200062, P. R. China

c NYU-ECNU Center for Computational Chemistry at New York University, East China Normal University, 3663 zhongshan Road, Shanghai 200062, P. R. China

Table of Contents

1. Experimental reactivity of 1-methoxynaphthalene catalyzed by Cu catalysts 2

2. Computational methods 3

3. The formation of three Cu carbenes 4

4. References 5

5. Cartesian Coordinate of Optimized Structure 6

1. Experimental reactivity of 1-methoxynaphthalene catalyzed by Cu catalysts

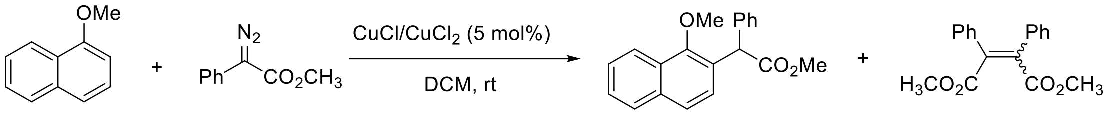

5 (3.0 equiv) **2** **6** trace **7**

Scheme S1 C(sp^2)-H bond functionalization of 1-methoxynaphthalene with α-diazoesters catalyzed by CuCl or $CuCl_2$

In a dried glass tube, copper catalyst (0.02 mmol, 5 mol%), 1-methoxynaphthalene **5** (189.6 mg, 1.2 mmol, 3 equiv) and DCM (1 mL) was added at room temperature. Then a solution of **2** (76.2 mg, 0.4 mmol) dissolved in 1 mL DCM was introduced into the reaction mixture by a syringe. The resulting mixture was continually stirred at room temperature until **6** was consumed completely determined by TLC analysis. After being filtrated through celite and concentrated, the residue was purified by column chromatography on silica gel to afford the desired product. The yield was determined by ^{1}H-NMR of crude product, using CH_2Br_2 as internal standard.

The reaction of 1-methoxynaphthalene **5** with the diazoester **2** in standard condition only delivered a trace amount of *ortho*-selective C-H bond functionalization products **6**. This result indicated that the hydroxyl was vital not only for site-selectivity but also for reactivity.[1]

2. Computational methods

All DFT calculations are performed using the Gaussian09 program package.[2] The geometric structures of intermediates and transition states are directly optimized in the solution phase by using the ωB97XD functional.[3] The SDD basis set[4] combined with the effective core potential is used to describe the metal element Cu, and the large 6-31+G** basis set[5] is utilized to describe the nonmetallic elements C, H, O, N and Cl. Frequency analyses are also performed at the same computational level to confirm that the intermediates are local minima and the transition states have only one imaginary frequency. The intrinsic reaction coordinate (IRC)[6] calculations are performed to make sure that all transition state structures connect the correct reactants and products in the forward and backward reaction directions. The solvent effect of dichloromethane is evaluated using the SMD[7] model with a dielectric constant $\varepsilon = 8.93$ in Gaussian09. All the calculated energies refer to the Gibbs free energies in the units of kcal mol^{-1} at the temperature of 298.15 K. More structure details about the intermediates and transition states are provided in Supporting Information.

3. The formation of three Cu carbenes

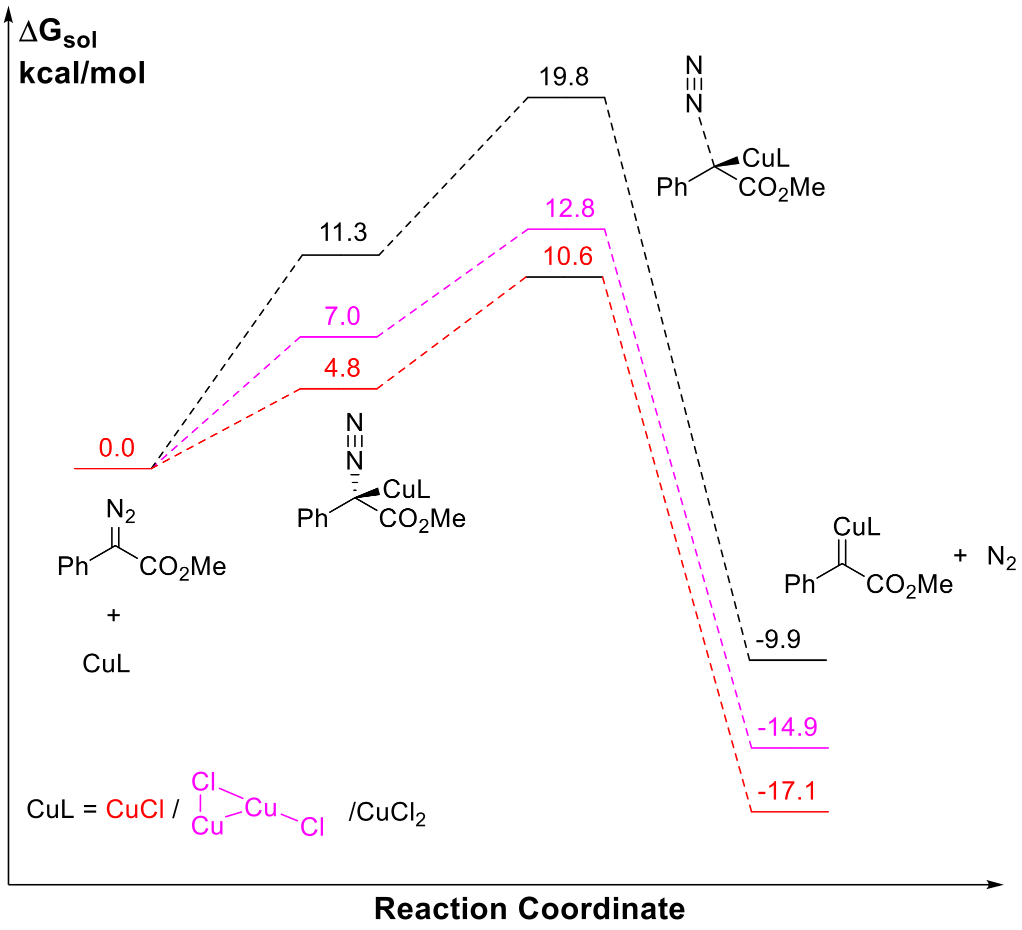

Figure S1 Calculated reaction pathways of diazoacetates and copper catalyst CuCl monomer, the $(CuCl)_2$ dimer and $CuCl_2$ monomer. Denoted by the red, pink and black lines respectively, to generate the products N_2 and Cu carbenes

4. References

1 B. Ma, Z. Tang, J. Zhang and L. Liu, Copper-catalysed *ortho*-selective C-H bond functionalization of phenols and naphthols with α-aryl-α-diazoesters, *Chem. Commun.*, 2020, **56**, 9485-9488.

2 M. J. Frisch, G. W. Trucks, H. B. Schlegel, G. E. Scuseria, M. A. Robb, J. R. Cheeseman, G. Scalmani, V. Barone, B. Mennucci, G. A. Petersson, H. Nakatsuji, M. Caricato, X. Li, H. P. Hratchian, A. F. Izmaylov, J. Bloino, G. Zheng, J. L. Sonnenberg, M. Hada, M. Ehara, K. Toyota, R. Fukuda, J. Hasegawa, M. Ishida, T. Nakajima, Y. Honda, O. Kitao, H. Nakai, T. Vreven, J. A. Montgomery, Jr., J. E. Peralta, F. Ogliaro, M. Bearpark, J. J. Heyd, E. Brothers, K. N. Kudin, V. N. Staroverov, T. Keith, R. Kobayashi, J. Normand, K. Raghavachari, A. Rendell, J. C. Burant, S. S. Iyengar, J. Tomasi, M. Cossi, N. Rega, J. M. Millam, M. Klene, J. E. Knox, J. B. Cross, V. Bakken, C. Adamo, J. Jaramillo, R. Gomperts, R. E. Stratmann, O. Yazyev, A. J. Austin, R. Cammi, C. Pomelli, J. W. Ochterski, R. L. Martin, K. Morokuma, V. G. Zakrzewski, G. A. Voth, P. Salvador, J. J. Dannenberg, S. Dapprich, A. D. Daniels, O. Farkas, J. B. Foresman, J. V. Ortiz, J. Cioslowski, and D. J. Fox, *Gaussian 09*, *Revision B.01*, Gaussian, Inc., Wallingford CT, 2010.

3 (a) L. A. Burns, A. Vazquez-Mayagoitia, B. G. Sumpter and C. D. Sherrill, Density-functional approaches to noncovalent interactions: a comparison of dispersion corrections (DFT-D), exchange-hole dipole moment (XDM) theory, and specialized functionals, *J. Chem. Phys.*, 2011, **134**, 084107; (b) J. D. Chai and M. Head-Gordon, Long-range corrected hybrid density functionals with damped atom-atom dispersion corrections, *Phys. Chem. Chem. Phys.*, 2008, **10**, 6615-6620.

4 M. Dolg, U. Wedig, H. Stoll and H. Preuss, Energy-adjusted ab initio pseudopotentials for the first row transition elements, *J. Chem. Phys.*, 1987, **86**, 866-872.

5 W. J. Hehre, R. Ditchfield and J. A. Pople, Self-Consistent Molecular Orbital Methods. XII. Further Extensions of Gaussian-Type Basis Sets for Use in Molecular Orbital Studies of Organic Molecules, *J. Chem. Phys.*, 1972, **56**, 2257-2261.

6 (a) K. Fukui, The path of chemical reactions-the IRC approach, *Acc. Chem. Res.*, 1981, **14**, 363-368; (b) K. Fukui, A Formulation of the reaction coordinate, *J. Phys. Chem.*, 1970, **74**, 4161-4163.

7 V. Marenich, C. J. Cramer and D. G. Truhlar, Universal solvation model based on solute electron density and on a continuum model of the solvent defined by the bulk dielectric constant and atomic surface tensions, *J. Phys. Chem. B*, 2009, **113**, 6378-6396.

5. Cartesian Coordinate of Optimized Structure

Structure and coordinates of 1-naphthol.log

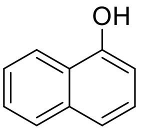

C	2.36843800	-1.07038100	0.00001500
C	1.04261200	-1.43036800	0.00001000
C	0.02765900	-0.43713600	-0.00001000
C	0.39252400	0.93938600	-0.00001200
C	1.77454500	1.27465800	-0.00001000
C	2.73819500	0.29662400	0.00000200
H	3.13875500	-1.83551800	0.00003300
H	0.75873400	-2.47726200	0.00002200
C	-1.36077200	-0.77068700	-0.00001200
C	-0.62029500	1.93792300	0.00000100
H	2.05415200	2.32484400	-0.00001700
H	3.78986700	0.56740700	0.00000800
C	-1.94321600	1.57444400	0.00001700
C	-2.32356600	0.21138000	0.00000000
H	-0.33102800	2.98487000	0.00000200
H	-2.71840400	2.33480300	0.00003100
H	-3.37603000	-0.05973700	-0.00000200
O	-1.65940200	-2.09687500	-0.00004200
H	-2.61757200	-2.21947400	0.00025600

Structure and coordinates of React-biCuCl.log

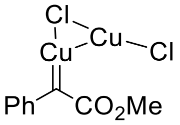

C	-2.63109900	-2.95933400	-0.70109700
C	-1.76322700	-1.88323200	-0.70230400
C	-2.11377400	-0.66813000	-0.05196000
C	-3.37262600	-0.58253000	0.60829900
C	-4.23076000	-1.66462700	0.61196900
C	-3.86047100	-2.84653200	-0.04384500
H	-2.36381500	-3.88293900	-1.20279900
H	-0.80153700	-1.94067400	-1.20418500
H	-3.65884300	0.33253200	1.11848600
H	-5.18873600	-1.60192600	1.11642100

H	-4.54356300	-3.69093400	-0.04082800
C	-1.57437900	1.67469900	0.57419400
O	-1.29281500	1.87450600	1.74048800
O	-2.16143000	2.54111400	-0.24028500
C	-2.47556600	3.83711800	0.30677900
H	-2.89446600	4.40264300	-0.52364500
H	-3.20974100	3.74054300	1.10939700
H	-1.56964200	4.31844400	0.67961600
C	-1.20553400	0.41414200	-0.10917500
Cu	0.48683300	0.39843600	-0.90750900
Cl	2.46611300	0.30135500	-1.91582400
Cu	2.82338600	-0.33337300	0.17727200
Cl	3.13580300	-0.93776300	2.21968600

Structure and coordinates of 1-TS-o1.log

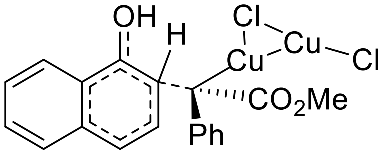

C	-2.86077900	0.60397600	-2.32424700
C	-1.62787800	0.51152000	-1.69719100
C	-1.35196400	1.24481600	-0.52180600
C	-2.37645800	2.05715600	0.01137300
C	-3.60872300	2.15660200	-0.62164300
C	-3.85387900	1.42720400	-1.78541100
H	-3.05482400	0.03608200	-3.22855500
H	-0.85316400	-0.13126700	-2.10701700
H	-2.19662600	2.61891800	0.92229800
H	-4.38228500	2.79315100	-0.20362300
H	-4.81975000	1.50146500	-2.27627600
C	0.38110600	2.21062900	1.04692300
O	0.83120800	2.09045200	2.17052100
O	0.33061000	3.39140900	0.40806500
C	0.93059800	4.50669600	1.08241800
H	0.80252400	5.35291000	0.40852300
H	0.42682600	4.69814700	2.03245000
H	1.99269100	4.31943600	1.25816700
C	-0.04594100	1.13230700	0.09793600
Cu	1.52088800	0.60027500	-0.87119800
Cl	3.41786800	0.28706900	-2.02057100
Cu	3.46756700	-1.02495800	-0.23487200
Cl	3.13471000	-2.24035800	1.52981200
C	-2.30054400	-2.71623000	-0.62282100
C	-1.98790100	-1.80120100	0.41111200

C	-3.60823600	-2.90109800	-1.01035000
C	-0.63920500	-1.54555200	0.79789200
C	-3.02666700	-1.08698800	1.06862200
C	-4.64190700	-2.18760800	-0.36741300
H	-3.84742700	-3.59419300	-1.81054000
C	-0.34360900	-0.49936900	1.68983800
C	-2.70910300	-0.17704800	2.13239500
C	-4.35872000	-1.30519300	0.65418400
H	-5.67137600	-2.33988100	-0.67779600
C	-1.41244700	0.08409900	2.44409900
H	0.67500000	-0.38618400	2.04363100
H	-3.52272000	0.30176800	2.66858800
H	-5.15904100	-0.76291700	1.14954900
H	-1.15709500	0.78831800	3.22722800
H	-1.49749300	-3.25496600	-1.11423900
O	0.29853800	-2.29889500	0.24003100
H	1.17643300	-2.17110900	0.66211600

Structure and coordinates of 1-TS-p1.log

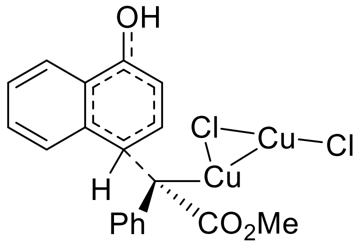

C	0.64818800	-1.71523500	2.36791400
C	0.12523300	-0.78030700	1.47898700
C	0.86976500	0.35065300	1.08960200
C	2.16775400	0.51188500	1.63054700
C	2.68294400	-0.41296500	2.52175800
C	1.92796600	-1.53640100	2.88466000
H	0.05574900	-2.57995700	2.65045200
H	-0.87667900	-0.91250300	1.08251800
H	2.76998600	1.36846500	1.34440600
H	3.67863300	-0.27396800	2.93149200
H	2.34014100	-2.26401300	3.57762600
C	0.95169600	2.61637200	-0.02850000
O	1.36823000	3.15385300	-1.03912600
O	0.90155300	3.24735900	1.15613300
C	1.31256700	4.62123800	1.16848600
H	1.20377000	4.94505100	2.20303600
H	2.35330000	4.71569300	0.85034100
H	0.67117500	5.21985400	0.51697200

C	0.30838500	1.27545300	0.12108100
Cu	-1.57248800	1.40515200	-0.23302000
Cl	-3.75653800	1.45392900	-0.61397600
Cu	-3.46007600	-0.64728000	0.02026600
Cl	-3.17103600	-2.70383100	0.61732700
C	3.58058800	-2.14746600	-0.58061000
C	2.36130400	-1.58000700	-1.01384200
C	4.73249300	-1.39074700	-0.58911900
C	1.14321800	-2.33912200	-1.03282500
C	2.31727200	-0.23078400	-1.44570300
C	4.69592800	-0.05054200	-1.02292000
H	5.66981600	-1.82616100	-0.25766600
C	-0.03739200	-1.80796600	-1.54495700
C	1.05591300	0.35419700	-1.81101000
C	3.51073800	0.52068000	-1.44057100
H	5.60946000	0.53662200	-1.02747100
C	-0.06074000	-0.48381200	-1.95792100
H	-0.93231400	-2.42122700	-1.59089800
H	1.05429500	1.33158500	-2.27813300
H	3.47879500	1.55719100	-1.76242400
H	-0.97840900	-0.07076400	-2.36715500
H	3.60239300	-3.17864800	-0.24539300
O	1.21795600	-3.59010000	-0.57742600
H	0.34816300	-4.01680000	-0.60179000

Structure and coordinates of 1-Int-p2.log

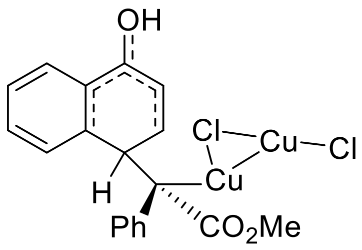

C	0.33309500	-1.46957300	2.67072600
C	-0.02538400	-0.71195800	1.55587500
C	0.75288000	0.36610100	1.11645600
C	1.91471100	0.66351700	1.84859800
C	2.27449000	-0.08105000	2.96895800
C	1.48793200	-1.15743300	3.38383800
H	-0.29928800	-2.29728300	2.98006200
H	-0.93267800	-0.96575500	1.01434900
H	2.55135400	1.48578000	1.53645200
H	3.17825200	0.17496300	3.51527000
H	1.77081400	-1.74031200	4.25572900

C	0.78213300	2.51724500	-0.25746500
O	1.13900600	3.11535700	-1.26994100
O	0.62923400	3.16620700	0.91612100
C	0.88809700	4.57001300	0.90747000
H	0.69262200	4.90835000	1.92536000
H	1.92908800	4.77306700	0.64177400
H	0.22511400	5.08592600	0.20801200
C	0.41520200	1.08684900	-0.17425900
Cu	-1.55751300	1.29249600	-0.38162300
Cl	-3.76669100	1.39274200	-0.61281400
Cu	-3.46118200	-0.72062500	-0.03269500
Cl	-3.17626800	-2.80986300	0.41988200
C	3.99899000	-1.92988500	-0.59865700
C	2.69480400	-1.51082800	-0.93419000
C	5.02209200	-1.00411400	-0.55019500
C	1.62341200	-2.46184000	-1.05642300
C	2.41515500	-0.15382600	-1.19971700
C	4.75498300	0.34047700	-0.84566900
H	6.02946000	-1.31688000	-0.29682100
C	0.34058800	-2.09920800	-1.53920900
C	1.00586100	0.30651500	-1.42716100
C	3.47057600	0.76052100	-1.16254600
H	5.56239300	1.06619200	-0.82304200
C	0.06751100	-0.78672400	-1.74413700
H	-0.40647500	-2.86930200	-1.70336900
H	0.98163700	1.04364400	-2.23996700
H	3.27264000	1.80649600	-1.37130600
H	-0.92011300	-0.49941900	-2.09684300
H	4.19379200	-2.97670400	-0.39320800
O	1.89627200	-3.70130400	-0.74264400
H	1.12495000	-4.28283100	-0.85942500

Structure and coordinates of 1-Int-o2.log

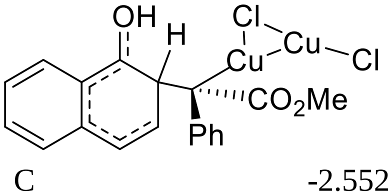

C	-2.55268600	1.04992100	-2.46472000
C	-1.43125200	0.74728500	-1.69392100
C	-1.21234200	1.35163000	-0.44781200
C	-2.15961300	2.28782900	-0.00732600
C	-3.27571100	2.60704400	-0.77718100
C	-3.48182200	1.98275400	-2.00760300
H	-2.69526500	0.55926900	-3.42331200

H	-0.70869300	0.02157600	-2.06295500
H	-2.02390800	2.77473100	0.95375300
H	-3.99040100	3.33865300	-0.41063500
H	-4.35589800	2.22530200	-2.60518300
C	0.59364800	1.99781700	1.23114800
O	1.11160500	1.84520600	2.33294200
O	0.60627300	3.19634100	0.60988600
C	1.28767100	4.25415200	1.28517800
H	1.17866100	5.12774500	0.64229400
H	0.83526000	4.44897500	2.26093200
H	2.34673700	4.01612500	1.41599000
C	-0.03675900	0.93212300	0.41438400
Cu	1.53696200	0.57911800	-0.75077100
Cl	3.31854700	0.17706500	-2.05117900
Cu	3.35005500	-1.15600300	-0.28035800
Cl	2.89782100	-2.42332400	1.42365000
C	-2.49034300	-2.67548800	-0.69137300
C	-2.15031300	-1.74711400	0.31685200
C	-3.81659200	-2.89899900	-0.99622400
C	-0.79011800	-1.45918600	0.62736600
C	-3.15880100	-1.06760100	1.05031800
C	-4.81782700	-2.21936600	-0.28190300
H	-4.08758100	-3.59514900	-1.78240700
C	-0.42039100	-0.26651300	1.42110400
C	-2.79688100	-0.15813800	2.12613800
C	-4.49753300	-1.32744000	0.73065400
H	-5.86124500	-2.40032400	-0.52161900
C	-1.51857800	0.19030000	2.32518700
H	0.49328900	-0.46751900	1.98888000
H	-3.59477800	0.24558200	2.74187300
H	-5.28322200	-0.82085900	1.28245700
H	-1.23957800	0.90099600	3.09530900
H	-1.70427600	-3.18480900	-1.23834900
O	0.10990800	-2.25006900	0.15243800
H	1.02432100	-2.09207900	0.50997100

Structure and coordinates of 1-TS-p3.log

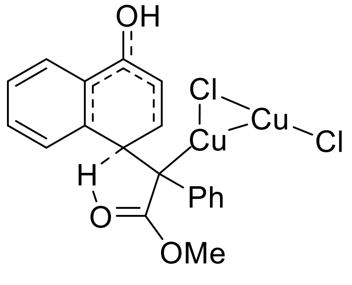

C	-2.26960900	2.88605100	-2.06416100
C	-1.65910200	1.78208700	-1.47702300
C	-0.93125600	1.89487300	-0.27756600
C	-0.84126600	3.17152400	0.30556500
C	-1.45458500	4.27531000	-0.28540800
C	-2.17300800	4.14534600	-1.47233300
H	-2.82169500	2.75824500	-2.99113700
H	-1.74970600	0.81364700	-1.96163900
H	-0.28934900	3.30821900	1.22691200
H	-1.36631500	5.24643300	0.19407400
H	-2.64787200	5.00869000	-1.92851900
C	0.19897800	0.55586600	1.65946700
O	0.20161700	-0.58017500	2.20762700
O	0.66419100	1.61549600	2.29825900
C	1.28029200	1.39436900	3.57875200
H	1.63353000	2.37385900	3.89850000
H	0.55147600	1.00321700	4.29136500
H	2.11864400	0.70169500	3.47937700
C	-0.29963200	0.66270500	0.29754600
Cu	1.50797600	0.77755000	-0.57355700
Cl	3.47271900	0.94310400	-1.59010000
Cu	3.51251600	-0.87061700	-0.30447900
Cl	3.56608400	-2.64241000	0.92966700
H	-0.46157800	-1.14446600	1.08567300
C	-1.01211700	-0.67397900	0.03458500
C	-2.44007100	-0.79542700	0.37404300
C	-0.51882400	-1.57004000	-0.95582500
C	-3.06291800	0.07619200	1.29125300
C	-3.21622400	-1.84371200	-0.17856800
C	-1.26491400	-2.59981000	-1.49154600
H	0.51924200	-1.46021800	-1.26227800
C	-4.38985600	-0.08603200	1.63060000
H	-2.49854900	0.89194200	1.72929900
C	-4.57612900	-2.00059000	0.18297900
C	-2.60036500	-2.73697800	-1.11491100
H	-0.82644400	-3.29310800	-2.20237400
C	-5.15557800	-1.13050600	1.07665100
H	-4.84872500	0.60238100	2.33364800
H	-5.15057500	-2.81306100	-0.24780600
H	-6.19778000	-1.24764200	1.35484700
O	-3.36870900	-3.70326800	-1.60218600
H	-2.87791800	-4.26262000	-2.22376700

Structure and coordinates of 1-TS-o3.log

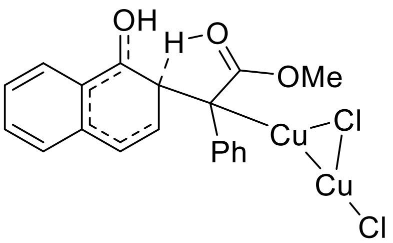

C	-0.53422400	3.95151200	-1.65335100
C	-0.59410700	2.68677500	-1.07820800
C	0.25478700	2.32310000	-0.01378900
C	1.17300200	3.28532400	0.44201100
C	1.23149100	4.55103400	-0.14083800
C	0.38169100	4.89646600	-1.18893200
H	-1.20656700	4.19799300	-2.47025600
H	-1.31159000	1.96561200	-1.46145700
H	1.85244800	3.04719500	1.25064300
H	1.95222500	5.27202700	0.23495700
H	0.43315800	5.88271300	-1.63986800
C	0.74971600	0.53647500	1.82201600
O	0.14622400	-0.29311700	2.54721700
O	1.92382400	1.01725100	2.17546100
C	2.52588400	0.48733200	3.37187300
H	3.47757400	1.00843900	3.46431300
H	1.89325700	0.69078100	4.23765000
H	2.68803300	-0.58677900	3.26293400
C	0.13228900	0.93105600	0.55230300
Cu	1.49331900	0.26615800	-0.77170000
Cl	3.13459000	-0.58261400	-2.03891900
Cu	2.45591900	-2.09256600	-0.56519300
Cl	1.81268900	-3.55655800	0.87216900
C	-3.69731500	-2.07944000	-0.90475800
C	-3.21605700	-0.94196500	-0.21471200
C	-5.05278300	-2.29907900	-1.00913900
C	-1.80946800	-0.68182400	-0.11339500
C	-4.12578000	-0.02346700	0.36947100
C	-5.96062300	-1.38925300	-0.43113600
H	-5.42367300	-3.17432000	-1.53220600
C	-1.30927200	0.40723400	0.66539000
C	-3.63456800	1.13222500	1.07286100
C	-5.50814000	-0.27480600	0.24543700
H	-7.02772800	-1.56935400	-0.51962200
C	-2.30749700	1.34461300	1.19089000
H	-0.99231000	-0.24995100	1.71185400
H	-4.35129100	1.82567100	1.50176000
H	-6.21147900	0.42292400	0.69005000

H	-1.93073300	2.21011500	1.72748700
H	-2.99270400	-2.77722700	-1.34306400
O	-1.01979800	-1.54874600	-0.70055400
H	-0.07641300	-1.29705500	-0.59147800

Structure and coordinates of 1-Int-o4.log

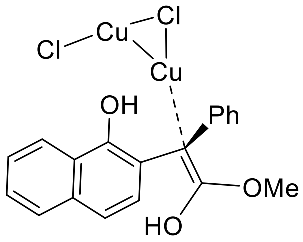

C	0.12705200	4.42186000	-0.68147900
C	0.43744200	3.08823500	-0.42956500
C	-0.42405700	2.27275500	0.31781900
C	-1.61166700	2.83598200	0.80500900
C	-1.92505500	4.17008300	0.55224200
C	-1.05891100	4.96869500	-0.19263300
H	0.80816800	5.02974700	-1.26964900
H	1.35982700	2.67072300	-0.82644700
H	-2.30069400	2.22518300	1.38098300
H	-2.85193600	4.58382100	0.93839200
H	-1.30726200	6.00606100	-0.39562000
C	1.01699200	0.49504000	1.33019000
O	1.16722400	-0.69726900	1.89524500
O	1.87910900	1.46352200	1.61562600
C	3.11995100	1.16731200	2.27282400
H	3.57766800	2.13768200	2.45780600
H	2.94757800	0.65273800	3.22095600
H	3.76727100	0.57499800	1.62128600
C	-0.13326700	0.81100500	0.58605600
Cu	1.08740900	0.08881400	-0.95069300
Cl	2.47503600	-0.59924300	-2.55876800
Cu	3.15191000	-1.53458600	-0.67798500
Cl	3.71355500	-2.31654200	1.26941400
C	-4.32326200	-1.02911100	-1.42741900
C	-3.41800800	-0.94548800	-0.33565200
C	-5.47726300	-1.76810400	-1.31976000
C	-2.21745700	-0.17435400	-0.41335400
C	-3.71710700	-1.63482700	0.87045200
C	-5.77711500	-2.45866000	-0.12066800
H	-6.16693700	-1.82163100	-2.15684700
C	-1.33745300	-0.08985000	0.65562200
C	-2.82350200	-1.52085700	1.96898100

C	-4.91593600	-2.39300900	0.94768800
H	-6.69394800	-3.03615800	-0.04730800
C	-1.68726800	-0.76666800	1.85968500
H	-3.05957100	-2.02533600	2.90160200
H	-5.14109700	-2.91695600	1.87297200
H	-1.03257800	-0.67305800	2.71815100
H	-4.09635800	-0.49962400	-2.34618400
O	-2.02548400	0.46357500	-1.59805700
H	-1.32340700	1.12435600	-1.52705100
H	2.07556400	-1.07568800	1.82892300

Structure and coordinates of 1-Int-p4.log

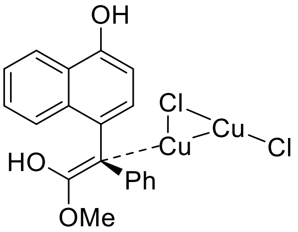

C	-0.13637800	4.50719200	0.11554400
C	0.19349500	3.17395700	0.34439900
C	-0.65606200	2.13226900	-0.07346900
C	-1.83165700	2.49103400	-0.75357400
C	-2.16031100	3.82612900	-0.97939900
C	-1.31783600	4.84524400	-0.54243400
H	0.54302700	5.28485100	0.45277400
H	1.12651600	2.95054000	0.84617400
H	-2.50782700	1.72249700	-1.11111100
H	-3.08347200	4.06371300	-1.50024400
H	-1.57237300	5.88632000	-0.71726800
C	0.38491600	0.21687100	1.24602000
O	0.36047600	-1.07358700	1.56957400
O	0.95828500	1.07203900	2.08349600
C	1.79132900	0.60077500	3.15428000
H	2.00752000	1.48572100	3.75053500
H	1.26580200	-0.13835700	3.76264900
H	2.72052900	0.18566500	2.75630300
C	-0.34699300	0.67163900	0.13791600
Cu	1.45699300	0.48649000	-0.84420900
Cl	3.33714700	0.18082600	-2.01812100
Cu	3.36493000	-1.17500100	-0.26241800
Cl	3.16738300	-2.38337700	1.53060000
C	-4.68081000	-1.80012200	0.48739700
C	-3.48721400	-1.52607700	-0.23074100
C	-4.93318800	-1.18046700	1.68716300

C	-3.22099800	-2.14877900	-1.48844600
C	-2.53438600	-0.60994600	0.29922900
C	-3.99934900	-0.26035400	2.21596400
H	-5.85083300	-1.39203800	2.22778600
C	-2.07184300	-1.85804400	-2.18071000
C	-1.32907400	-0.32622000	-0.42987300
C	-2.83479300	0.01492800	1.54126500
H	-4.20469600	0.23444700	3.16071700
C	-1.13852400	-0.94319100	-1.64334400
H	-2.13732000	0.73320500	1.96039400
H	-0.24028400	-0.73530500	-2.22212900
H	-5.39428800	-2.50417500	0.07288900
O	-4.15948900	-3.01787500	-1.94315300
H	-3.87936500	-3.39239400	-2.78826500
H	1.25368700	-1.45056900	1.74634100
H	-1.87550000	-2.32958800	-3.13997000

Structure and coordinates of 1-Int-p5.log

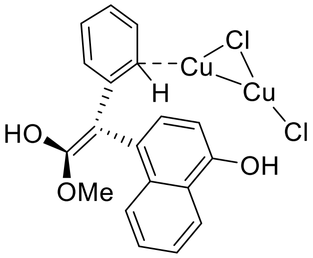

C	-2.44360900	1.41729900	2.56840400
C	-1.55021200	1.90058700	1.61024000
C	-0.31184800	1.27533100	1.37209900
C	-0.01525700	0.12334500	2.16144700
C	-0.92169600	-0.35069100	3.13392000
C	-2.15021700	0.29448000	3.33364000
H	-3.38553800	1.93798300	2.71407100
H	-1.82122000	2.78125900	1.04595300
H	0.97691500	-0.31758700	2.11625400
H	-0.62484000	-1.17378100	3.77859900
H	-2.84426500	-0.06799500	4.08444000
C	0.69441400	1.73624300	0.39885700
C	0.52335700	2.75402700	-0.48330100
O	1.45837100	2.98002400	-1.42676200
O	-0.52919400	3.57946000	-0.46481600
C	-1.04724100	4.06394900	-1.71651700
H	-2.08486100	4.33177000	-1.51980300
H	-0.50358600	4.95654500	-2.04203600
H	-1.01138000	3.28591700	-2.48154500

C	3.53066400	-2.04890700	-1.23666600
C	3.39035200	-0.87803100	-0.44501400
C	2.49305500	-2.49074100	-2.02194200
C	4.45404000	-0.40005300	0.37875900
C	2.16275300	-0.15552300	-0.46551000
C	1.27514100	-1.77210400	-2.05678800
H	2.60747100	-3.39058500	-2.61869000
C	4.29247300	0.73180900	1.14108800
C	2.00278900	1.01120200	0.34866200
C	1.11890400	-0.63350600	-1.30399400
H	0.46047300	-2.11989900	-2.68566600
C	3.06044100	1.42421300	1.12386900
H	0.18740200	-0.07513900	-1.35007200
H	2.94961700	2.30562100	1.74966700
H	4.47045700	-2.59044400	-1.21234700
Cu	-1.11351200	-1.37753500	1.15068600
Cl	-2.18002600	-2.92896800	-0.04643100
Cu	-2.47803800	-1.03097600	-1.12989700
Cl	-2.73958300	0.82273100	-2.18466000
O	5.60591300	-1.12110300	0.35856600
H	6.25734400	-0.71910400	0.94762900
H	1.41692600	3.89153000	-1.74864600
H	5.10684200	1.09211200	1.76442100

Structure and coordinates of 1-Int-o5.log

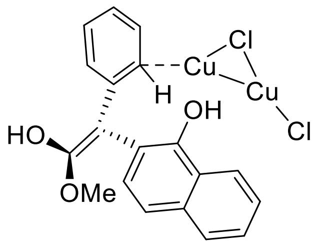

C	-3.35356100	1.16892600	0.69393200
C	-2.27220300	1.66723100	-0.03939000
C	-0.97997400	1.68582900	0.49369600
C	-0.79217000	1.16234500	1.80087500
C	-1.88465100	0.65310400	2.53471600
C	-3.17355600	0.65493000	1.97183700
H	-4.34104300	1.16628000	0.24325800
H	-2.43808400	2.04047500	-1.04427600
H	0.17535900	1.27103800	2.28629900
H	-1.74079600	0.34627100	3.56747100
H	-4.01353600	0.26675300	2.53805800
C	0.18499000	2.21155800	-0.25053500
C	0.11907100	3.28884500	-1.05908100

O	1.16984800	3.64072400	-1.82708900
O	-0.94439800	4.10559400	-1.23671300
C	-1.36817800	4.86817400	-0.09551200
H	-2.09311100	5.59034400	-0.47111000
H	-1.84368700	4.22972400	0.65352500
H	-0.51448600	5.39247200	0.34455800
C	3.16341600	-1.70264000	-1.12242200
C	2.90723900	-0.45175900	-0.49690800
C	4.33524300	-2.38061400	-0.88746700
C	1.69664200	0.28244900	-0.70172600
C	3.89798800	0.10118000	0.36572900
C	5.31307700	-1.83990400	-0.01890400
H	4.51366200	-3.33421100	-1.37481200
C	1.48590500	1.50244600	-0.08639700
C	3.67421800	1.36532600	0.97409900
C	5.09814000	-0.62740500	0.58835000
H	6.23624900	-2.38335800	0.15868300
C	2.50288900	2.03891700	0.74436100
H	4.43907400	1.78411800	1.62185700
H	5.84752400	-0.20172300	1.25054300
H	2.32510900	3.00349200	1.21239700
H	2.44215300	-2.14074900	-1.80693200
Cu	-0.79457100	-0.93330100	1.45464100
Cl	-0.40709400	-3.01630700	0.78774600
Cu	-1.89022000	-2.32864300	-0.68988400
Cl	-3.34519900	-1.70578500	-2.14810700
O	0.71850000	-0.17322400	-1.52668900
H	0.96169300	4.48001500	-2.26144700
H	0.81307900	-1.11738500	-1.70149800

Structure and coordinates of 1-TS-o6-2w.log

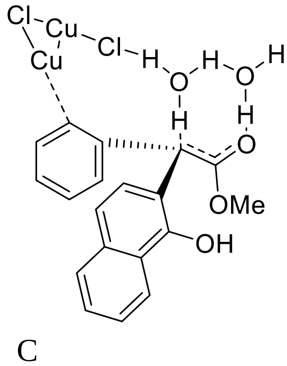

C	2.75676900	1.51653800	-1.94268400
C	1.74098100	1.92589800	-1.07493700
C	0.46531000	1.35949700	-1.13307700
C	0.23517500	0.32770500	-2.07569200
C	1.27515700	-0.11187800	-2.91943600

C	2.53772700	0.49477100	-2.85824900
H	3.73290900	1.98672500	-1.87490300
H	1.95578700	2.68882200	-0.33261400
H	-0.77335500	-0.05211600	-2.22739600
H	1.06937200	-0.86963900	-3.66961300
H	3.33206000	0.16243900	-3.51838700
C	-0.58384600	1.73471700	-0.12028700
C	-0.85993400	3.14119900	0.05909600
O	-1.57639700	3.59753600	1.02175900
O	-0.34149300	4.00901400	-0.75444800
C	-0.46509700	5.42061400	-0.46044200
H	0.08171500	5.91752200	-1.25892900
H	-1.51553000	5.71311900	-0.47381200
H	-0.01503300	5.63618800	0.50955800
O	-1.75301500	2.04993600	2.95777600
H	-1.77340600	2.50096400	3.81125600
H	-1.73594100	2.92055100	1.81338400
O	0.36286500	0.85562200	2.13547700
H	0.01429800	1.44775400	1.01274800
H	-0.88506300	1.55019500	2.88795600
H	1.31775800	0.89233800	2.28871900
C	-2.72923300	-2.71793800	0.60323700
C	-2.83550800	-1.35423900	0.22082500
C	-3.81585500	-3.55339000	0.50305100
C	-1.72054700	-0.46767700	0.30998100
C	-4.07566500	-0.85871900	-0.26592900
C	-5.05350200	-3.06455000	0.01753900
H	-3.72729400	-4.59504700	0.79693000
C	-1.81671700	0.85555400	-0.08962000
C	-4.16789700	0.50604500	-0.65146200
C	-5.18012100	-1.74845100	-0.35650000
H	-5.90326800	-3.73677100	-0.05650800
C	-3.07197000	1.32361200	-0.56990900
H	-5.11138400	0.88911200	-1.02980700
H	-6.12735500	-1.36833400	-0.73027000
H	-3.15951200	2.35520600	-0.90063600
H	-1.77982700	-3.08535700	0.97783800
Cu	0.95025100	-1.30346400	-0.89875200
Cl	2.20310500	-3.08797600	-0.26526600
Cu	2.99818200	-1.37343600	0.87096700
Cl	3.77295200	0.30161200	1.99632500
O	-0.54449600	-1.00900100	0.73815100
H	-0.08554100	-0.35609600	1.38951800

Structure and coordinates of 1-TS-p6-2w.log

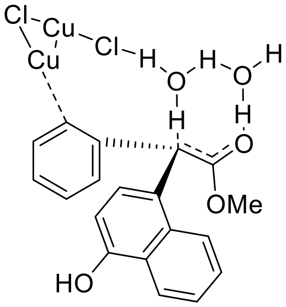

C	-2.43099500	0.76103500	2.65407800
C	-1.46291500	1.30112500	1.80695200
C	-0.22139600	0.66282400	1.57493200
C	-0.05609700	-0.61386900	2.19826500
C	-1.04448400	-1.15607200	3.05306500
C	-2.23933200	-0.46067400	3.29416300
H	-3.35684900	1.30875200	2.80556400
H	-1.66790100	2.25020500	1.33271100
H	0.90250100	-1.11926400	2.13238300
H	-0.83117800	-2.07952500	3.58550500
H	-2.98935600	-0.87053300	3.96206500
C	0.87205400	1.21613700	0.75881600
C	1.07339000	2.60804600	0.57392500
O	2.04795200	3.12607700	-0.04396000
O	0.12563300	3.43774200	1.07035600
C	0.25313900	4.82788200	0.77961000
H	-0.59437600	5.30420200	1.27404900
H	1.18910700	5.23189100	1.17242400
H	0.19753500	5.01269700	-0.29720500
O	1.64391200	3.29311600	-2.51544600
H	1.33390800	4.15725500	-2.82140900
H	1.93221100	3.36343100	-1.53659500
O	0.04406100	1.57366500	-2.07841700
H	0.22323800	1.38052900	-1.11168600
H	0.75165300	2.45861700	-2.40093700
H	-0.93087000	1.70231400	-2.17505900
C	3.32827800	-2.66064100	-1.36226500
C	3.31470200	-1.53143300	-0.49953200
C	2.19031300	-3.05929200	-2.01910700
C	4.49023700	-1.13191200	0.20022700
C	2.11165800	-0.78335000	-0.31997900
C	0.98514000	-2.34416500	-1.83017200
H	2.21128600	-3.92338600	-2.67638100
C	4.45901000	-0.04910900	1.04458200
C	2.09411800	0.36610900	0.54217200

C	0.95576100	-1.24232200	-1.01115000
H	0.07859800	-2.66561800	-2.33469000
C	3.26223700	0.67957900	1.20672300
H	0.01620700	-0.71587000	-0.87393600
H	3.27174500	1.53354600	1.87791800
H	4.25745300	-3.20495300	-1.49356000
Cu	-1.39433300	-1.76611400	0.99612900
Cl	-2.87800200	-2.49863300	-0.49135000
Cu	-2.92175900	-0.41296600	-1.21671700
O	5.60832700	-1.87762800	-0.01040200
H	6.34332900	-1.51707400	0.50204600
H	5.35301400	0.24828300	1.58757500
Cl	-2.99803300	1.59126000	-2.05939100

Structure and coordinates of 1-Int-o7.log

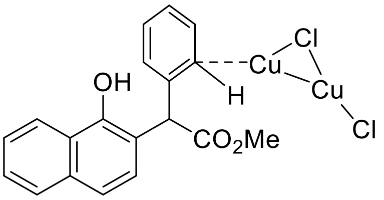

C	3.39745600	1.60705800	-0.85485600
C	2.34455200	1.75660600	0.04968800
C	1.03108500	1.46221900	-0.31788300
C	0.77921900	0.99888400	-1.62821600
C	1.84949300	0.83166500	-2.53599200
C	3.16095100	1.13743000	-2.14278900
H	4.40854300	1.85015300	-0.54358400
H	2.54720900	2.11111500	1.05634400
H	-0.24119400	0.87547100	-1.98137200
H	1.64146700	0.54115200	-3.56166300
H	3.97712700	1.01926900	-2.84778600
C	-0.08671400	1.68886200	0.69451400
C	-0.30001900	3.17208100	1.00440000
O	-0.72466300	3.57365400	2.06670500
O	0.00471700	3.96479400	-0.02116400
C	-0.19506000	5.37404200	0.17131500
H	0.11555200	5.84056600	-0.76241700
H	-1.24842500	5.58320300	0.37057500
H	0.41997500	5.73451800	0.99867700
H	0.25741500	1.28157100	1.65045700
C	-3.43094500	-2.06890100	0.77857800
C	-3.02398000	-0.78780100	0.32034000
C	-4.59054800	-2.64074500	0.31013800
C	-1.80698100	-0.18161100	0.77830500

C	-3.83304700	-0.09812500	-0.62239200
C	-5.39648700	-1.95801800	-0.63164200
H	-4.89148100	-3.62222800	0.66422600
C	-1.41466600	1.05729600	0.30407900
C	-3.42377400	1.18292300	-1.08272700
C	-5.02640000	-0.71473500	-1.08406300
H	-6.30871000	-2.42093300	-0.99628600
C	-2.25496400	1.72791000	-0.62873100
H	-4.04301700	1.71746900	-1.79709400
H	-5.64130400	-0.18311600	-1.80556400
H	-1.94310200	2.69941500	-1.00219700
H	-2.81331900	-2.59300900	1.49954400
Cu	1.32102600	-1.01762700	-1.35083200
Cl	1.90327100	-3.13172600	-0.93230300
Cu	2.01223700	-2.01623200	0.98001900
Cl	1.94234700	-0.75585500	2.74657200
O	-1.11705200	-0.92464900	1.67272900
H	-0.25592000	-0.55646200	1.93058200

Structure and coordinates of Product-o8.log

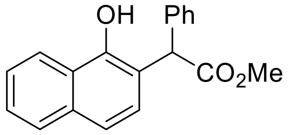

C	-3.59245700	-2.52083300	-0.28985800
C	-2.88326400	-1.47807700	-0.88559500
C	-1.93241600	-0.75837900	-0.15589100
C	-1.69810200	-1.10004500	1.17875300
C	-2.40728200	-2.13877900	1.77549300
C	-3.35624800	-2.85254900	1.04276200
H	-4.32422300	-3.07530600	-0.86970500
H	-3.06680900	-1.22451600	-1.92709300
H	-0.95782300	-0.55143800	1.75368200
H	-2.21660200	-2.39277000	2.81387900
H	-3.90315700	-3.66731000	1.50769500
C	-1.17774700	0.38476800	-0.83039600
C	-1.91343600	1.71698200	-0.72157900
O	-1.94246200	2.53696300	-1.61612200
O	-2.50080900	1.89076500	0.45917000
C	-3.16934000	3.14409800	0.66799400
H	-3.58624800	3.08515700	1.67217200
H	-2.45673000	3.96915800	0.59857200
H	-3.96570300	3.27795300	-0.06701500
H	-1.16357600	0.19114100	-1.90939400

C	3.49331500	-1.38269700	-0.64708000
C	2.54735400	-0.38244900	-0.29585200
C	4.80419800	-1.26844900	-0.25234300
C	1.17860500	-0.46917100	-0.69275900
C	2.96781500	0.73620900	0.47411600
C	5.22816600	-0.15494100	0.51319000
H	5.52151000	-2.03565000	-0.52727000
C	0.26727000	0.51680800	-0.35954800
C	2.01790600	1.72897600	0.82955800
C	4.33104700	0.82143700	0.86847400
H	6.26749000	-0.07655400	0.81796600
C	0.71459800	1.61283800	0.42296300
H	2.33395800	2.58002900	1.42554400
H	4.65015900	1.67749200	1.45700400
H	0.00755000	2.38396300	0.71267700
H	3.16901000	-2.23674700	-1.23133300
O	0.85262900	-1.56984500	-1.42111800
H	-0.10355400	-1.70216000	-1.45723300

Structure and coordinates of React-moCuCl.log

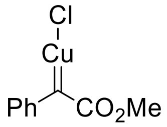

C	-2.53728800	-2.46947600	-0.17628800
C	-1.33790600	-1.78864400	-0.06833300
C	-1.31755600	-0.37455200	0.06844100
C	-2.55504100	0.32672500	0.09932000
C	-3.74919100	-0.35981400	-0.00871400
C	-3.73780700	-1.75371600	-0.14805800
H	-2.54812500	-3.54857200	-0.28476100
H	-0.39099400	-2.32046200	-0.08880100
H	-2.56608900	1.40719000	0.20918200
H	-4.69256600	0.17490800	0.01241500
H	-4.67995600	-2.28652700	-0.23646600
C	-0.06287700	0.28302300	0.14243100
C	-0.06672900	1.74935600	0.34724200
O	-0.05220400	2.23071700	1.46502600
O	-0.02662700	2.43473500	-0.78727100
C	0.06402900	3.86853900	-0.67244400
H	0.97135000	4.14727400	-0.13291500
H	0.10342800	4.23619600	-1.69627200
H	-0.81568400	4.26235600	-0.15969900
Cu	1.61494400	-0.51763200	0.05395500

Cl	3.54224200	-1.48666500	-0.12417700

Structure and coordinates of 1′-TS-o1.log

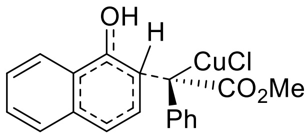

C	1.66844700	0.33254500	2.55639100
C	0.61163200	-0.02139000	1.73014800
C	0.07535000	0.89566400	0.80199500
C	0.65412100	2.18004500	0.72575300
C	1.70819500	2.53754400	1.55752700
C	2.22124200	1.61246200	2.46755200
H	2.06614700	-0.38656100	3.26550300
H	0.17883300	-1.01648600	1.79080300
H	0.26962300	2.90219500	0.01219700
H	2.14045400	3.53084400	1.48702700
H	3.05038200	1.88984700	3.11193400
C	-1.02561000	0.47900800	-0.05654600
C	-1.88040200	1.54731800	-0.66448500
O	-2.21489100	1.67503900	-1.82819900
O	-2.36836200	2.32066800	0.31905600
C	-3.38272400	3.26353300	-0.05312400
H	-4.24909900	2.74859300	-0.47505300
H	-3.65902300	3.76933300	0.87131600
H	-2.99232000	3.98352400	-0.77620900
Cu	-2.16108100	-1.00490500	0.37261300
Cl	-3.40632100	-2.71664200	0.81749700
C	0.71174400	0.99471000	-2.30674000
C	1.99409900	1.16532800	-1.89341700
C	2.65041800	0.14186500	-1.12969000
C	1.99981400	-1.10159400	-0.89857600
C	0.66949100	-1.26184700	-1.37679000
C	-0.02734600	-0.17861300	-1.93994700
H	4.44058100	1.28470900	-0.74815300
H	0.19367800	1.76962000	-2.85988400
C	3.93881900	0.33521800	-0.58575000
C	2.64986900	-2.11832800	-0.15727600
H	-0.98476600	-0.36334200	-2.41558100
C	3.90385900	-1.89704100	0.36363900
C	4.54716500	-0.65909800	0.15152500
H	2.14266500	-3.06188900	0.01171700
H	4.39731800	-2.67023700	0.94356700
H	5.53353000	-0.49074000	0.57348000

O	0.10739000	-2.45381500	-1.19319300
H	-0.80872400	-2.46143300	-1.51253500
H	2.53732000	2.07808800	-2.11781900

Structure and coordinates of 1′-TS-p1.log

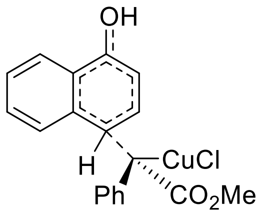

C	0.97964200	-1.42512800	2.67416900
C	0.04425900	-1.08115600	1.70209600
C	0.04423800	0.19835500	1.11213400
C	1.02373400	1.12528000	1.53653800
C	1.94924300	0.78517800	2.50879700
C	1.93497800	-0.49505600	3.07527100
H	0.95950900	-2.41518400	3.11876100
H	-0.71274200	-1.79737700	1.39631300
H	1.05656700	2.11516300	1.09363500
H	2.69253000	1.51042100	2.82490600
H	2.66652200	-0.76024500	3.83270100
C	-0.93266800	0.50363400	0.07619500
C	-1.16195800	1.93462000	-0.28805600
O	-1.15528600	2.44816400	-1.39318700
O	-1.52129400	2.61068600	0.81595700
C	-1.95090500	3.96484600	0.62087700
H	-2.85719300	3.99448100	0.01071400
H	-2.15713000	4.34914600	1.61922300
H	-1.16380100	4.55314600	0.14403200
Cu	-2.55112900	-0.49459600	-0.10177900
Cl	-4.34698100	-1.71557700	-0.16961500
C	-0.17929500	-1.44585800	-1.71504900
C	0.20603200	-0.09805500	-1.77526100
C	1.56560600	0.25074500	-1.46182600
C	2.42143800	-0.72106200	-0.88573700
C	1.91376100	-2.05121300	-0.71030000
C	0.64663200	-2.41295000	-1.15812900
H	1.40596200	2.30821300	-2.08386400
H	-1.16259800	-1.73109100	-2.07614400
C	2.06080000	1.55849100	-1.65094500
C	3.73841100	-0.37469500	-0.50578100
H	0.31063400	-3.44063100	-1.05704500
C	4.19509400	0.90987400	-0.70230700

C	3.35032800	1.87965500	-1.28006800
H	4.38157400	-1.12611500	-0.06103000
H	5.20669400	1.17705200	-0.41339900
H	3.72001100	2.88913800	-1.43365100
O	2.74000700	-2.92799700	-0.13574500
H	2.31878900	-3.79668800	-0.05732700
H	-0.38756400	0.59881800	-2.35470100

Structure and coordinates of 1′-Int-p2.log

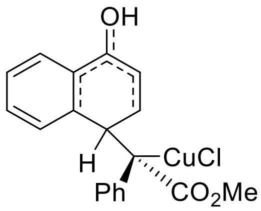

C	0.58325700	-1.32168400	2.93938700
C	-0.12612900	-1.04931500	1.76887800
C	-0.02722700	0.18995100	1.12185000
C	0.81220900	1.15553000	1.70270500
C	1.51589500	0.89504800	2.87517000
C	1.40993100	-0.34979400	3.49803800
H	0.47821800	-2.29145900	3.41786100
H	-0.77707800	-1.81277100	1.34931700
H	0.91803600	2.12645900	1.22877400
H	2.15470900	1.66450700	3.30003100
H	1.95886000	-0.55456000	4.41275300
C	-1.18195700	1.79703100	-0.49066800
O	-1.23282900	2.33264200	-1.59624500
O	-1.63389300	2.43553500	0.61042300
C	-2.21978100	3.72056500	0.40628900
H	-2.55324000	4.04952000	1.39105900
H	-1.48690300	4.42725000	0.00678700
H	-3.07234300	3.65740500	-0.27517800
C	-0.70751900	0.42645000	-0.21078200
Cu	-2.45487400	-0.53347800	-0.25747200
Cl	-4.27008200	-1.73659800	-0.27482300
C	3.92194500	-0.24599000	-0.55204800
C	2.59532200	-0.63193100	-0.83252300
C	4.29621500	1.07370300	-0.71155500
C	2.19213900	-2.01018300	-0.74370900
C	1.63792000	0.31550300	-1.25103000
C	3.35597700	2.01280700	-1.15951100
H	5.31609900	1.37896800	-0.50307100
C	0.91564600	-2.45512700	-1.16564700

C	0.20176600	-0.07689700	-1.41649600
C	2.04436300	1.64123900	-1.42124900
H	3.65532000	3.04702000	-1.30054000
C	-0.01252700	-1.53078100	-1.52680700
H	0.69923300	-3.51882000	-1.17799600
H	-0.22791900	0.41712400	-2.29632500
H	1.32016100	2.37955100	-1.74963900
H	-0.99491200	-1.85728300	-1.85710400
H	4.64304200	-0.98688000	-0.22529400
O	3.08661100	-2.85595400	-0.29927800
H	2.74767300	-3.76753100	-0.28371000

Structure and coordinates of 1′-Int-o2.log

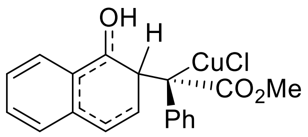

C	0.36640800	1.15479600	3.15212500
C	-0.20559400	0.46982400	2.07913900
C	-0.37448200	1.07984200	0.83008900
C	0.03955300	2.41451700	0.69668900
C	0.60310300	3.10744900	1.76488800
C	0.77543700	2.47729500	2.99838500
H	0.48490500	0.65337000	4.10838800
H	-0.53411100	-0.55731500	2.21621700
H	-0.08662900	2.92094400	-0.25627100
H	0.90983300	4.14133200	1.63296400
H	1.21886800	3.01428400	3.83181000
C	-1.86132300	0.99522000	-1.23668600
O	-1.98690100	0.81728500	-2.44509600
O	-2.65713200	1.85054200	-0.56397000
C	-3.69068600	2.48636000	-1.31810500
H	-4.18426800	3.16438800	-0.62186300
H	-3.27282200	3.04843900	-2.15689300
H	-4.40638400	1.74917400	-1.69222900
C	-0.89319600	0.29053300	-0.36163500
C	3.39425400	-1.88359400	0.32438600
C	2.60360100	-0.97604200	-0.42124300
C	4.75408500	-1.68808300	0.42156500
C	1.19497000	-1.13382500	-0.50445000
C	3.19985400	0.13782600	-1.07191300
C	5.34643800	-0.58626400	-0.22166600
H	5.36640700	-2.37639300	0.99370200
C	0.31344500	-0.19498000	-1.24876800

C	2.38703300	1.06685800	-1.83465100
C	4.58625400	0.30971100	-0.95613500
H	6.41824000	-0.43386600	-0.13870200
C	1.05855800	0.91585800	-1.91683400
H	-0.13218500	-0.80183600	-2.05643800
H	2.88920000	1.89136700	-2.33177700
H	5.05806300	1.15564600	-1.44600000
H	0.45602400	1.60762100	-2.49547300
H	2.92245400	-2.72616400	0.81765300
O	0.67520500	-2.15354400	0.09808500
H	-0.31498800	-2.09522000	0.07256800
Cu	-2.18324200	-1.15239000	0.15926600
Cl	-3.48907200	-2.80650300	0.65531800

Structure and coordinates of 1′-TS-p3.log

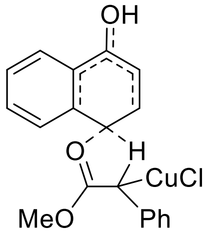

C	0.24626200	2.05949700	-2.86126000
C	0.13669500	1.07303500	-1.88629600
C	0.76373900	1.20366900	-0.63205300
C	1.50674300	2.37583100	-0.40246500
C	1.61445000	3.36207100	-1.38209600
C	0.98640500	3.21644600	-2.61746900
H	-0.25015000	1.92003900	-3.81765700
H	-0.43854400	0.17826700	-2.10912400
H	2.01152800	2.51870400	0.54454200
H	2.19569200	4.25535900	-1.16942400
H	1.07357000	3.98709300	-3.37744800
C	0.94165100	0.20474100	1.77322300
O	0.33024000	-0.54117300	2.59099300
O	1.86756800	1.04973100	2.19502000
C	2.21557800	1.00769700	3.58843800
H	2.98636400	1.76744600	3.71183200
H	1.34777000	1.24393800	4.20739100
H	2.61006200	0.02378400	3.85081100
C	0.60941200	0.09702600	0.36579700
Cu	2.18895800	-1.02638800	-0.19767600
H	-0.52033000	-1.00651200	1.55268600
C	-0.71209800	-0.69073900	0.33049100
C	-1.98094300	0.05472100	0.39911200

C	-0.76882300	-1.97675900	-0.27778100
C	-2.04470000	1.36854700	0.90774600
C	-3.19206500	-0.56007600	-0.00452800
C	-1.94503200	-2.58713200	-0.66427900
H	0.16630900	-2.51810300	-0.40073200
C	-3.24967400	2.03333700	1.00107100
H	-1.13411900	1.86967600	1.21715800
C	-4.42196200	0.13294000	0.10132200
C	-3.14560600	-1.89147200	-0.53381100
H	-1.93952800	-3.58995100	-1.07944300
C	-4.44950900	1.41530000	0.59784000
H	-3.27194100	3.04702300	1.38939300
H	-5.33753500	-0.35692000	-0.21101300
H	-5.39061700	1.94905800	0.68005000
O	-4.30709700	-2.42568700	-0.89186300
H	-4.18955800	-3.32347600	-1.23862800
Cl	3.81249600	-2.25030700	-0.94053000

Structure and coordinates of 1′-TS-o3.log

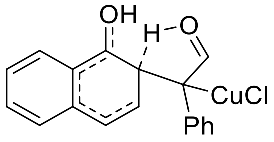

C	-0.32301100	2.57123500	-2.51641000
C	-0.26394300	1.40429800	-1.75966600
C	0.35628800	1.37635300	-0.49643000
C	0.92826500	2.57604200	-0.03381400
C	0.86724500	3.74223000	-0.79422200
C	0.23892200	3.75444000	-2.03846100
H	-0.81353900	2.55184500	-3.48565900
H	-0.70672900	0.49847300	-2.16486800
H	1.42800400	2.59967700	0.92588700
H	1.31662500	4.65065000	-0.40211700
H	0.19209000	4.66672500	-2.62550700
C	0.64975900	-0.02223900	1.69028500
O	0.18316200	-1.01812600	2.31725000
O	1.36011900	0.89806500	2.32520800
C	1.68648100	0.64507600	3.70081700
H	2.26339400	1.51178400	4.02095800
H	0.77763500	0.55148100	4.29828000
H	2.28821600	-0.26236500	3.78628000
C	0.37385400	0.09248500	0.27185200
Cu	2.20098300	-0.62578300	-0.23801300
H	-0.57382700	-1.40679300	1.15946000

C	-0.79462200	-0.87653100	0.03310200
C	-2.17245700	-0.36617000	0.23065100
C	-0.66281600	-2.01532300	-0.82456400
C	-2.42892200	0.80817000	0.96110900
C	-3.27692300	-1.10537200	-0.25617800
C	-1.78054900	-2.75087500	-1.28068100
C	-3.72730400	1.23085700	1.18186800
H	-1.60739000	1.40221400	1.34532900
C	-4.59771700	-0.66250900	-0.01546800
C	-3.03853500	-2.29835900	-1.00242200
H	-1.62658700	-3.65265100	-1.86566100
C	-4.82322500	0.49561100	0.69572100
H	-3.89996100	2.14835500	1.73628000
H	-5.42686400	-1.24998300	-0.39976100
H	-5.83556200	0.84029500	0.87978500
Cl	4.21381100	-1.29284100	-0.69846800
H	-3.89602500	-2.85873800	-1.36579900
O	0.56053100	-2.40762100	-1.13209600
H	0.56197800	-3.24000600	-1.63198100

Structure and coordinates of 1′-Int-o4.log

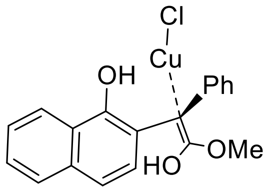

C	1.69389700	2.46814100	-2.56549600
C	1.31254900	1.35313600	-1.82059100
C	1.20626500	1.42077900	-0.42677400
C	1.47658700	2.64299900	0.20326300
C	1.85955100	3.75812900	-0.53819800
C	1.97158900	3.67461100	-1.92616000
H	1.77767000	2.38797300	-3.64520100
H	1.10153200	0.41913800	-2.33470800
H	1.38520800	2.72689400	1.28208100
H	2.06616200	4.69548600	-0.02986400
H	2.27054900	4.54414300	-2.50397600
C	0.69745600	0.23831900	0.37263800
C	1.36596400	-0.09280000	1.57058500
O	0.83966400	-0.83823400	2.53446400
O	2.55156500	0.39753100	1.85575000
C	3.32781900	-0.20837200	2.90987700
H	3.47563400	-1.27070900	2.70249600
H	4.28087400	0.31631400	2.88727000

H	2.83948800	-0.06651000	3.87495400
Cu	1.78709100	-1.36115800	-0.33535500
Cl	2.92768900	-2.98870900	-1.17615800
C	-1.42247900	1.04118400	1.44649300
C	-2.77666300	1.16245000	1.58225000
C	-3.64277300	0.43258400	0.72187900
C	-3.08044300	-0.41317700	-0.27035700
C	-1.65433500	-0.51046300	-0.38338900
C	-0.81863700	0.19259400	0.46727300
H	-5.48210000	1.18111900	1.57778000
H	-0.76817700	1.61916000	2.09440700
C	-5.05609000	0.52877100	0.82014800
C	-3.94098700	-1.14296700	-1.13215700
C	-5.30626000	-1.03069200	-1.01083500
C	-5.86996700	-0.18508900	-0.02652400
H	-3.51072400	-1.79174200	-1.88700400
H	-5.95637100	-1.59438500	-1.67306600
H	-6.94940400	-0.10147800	0.05809000
O	-1.22518200	-1.31685000	-1.37972300
H	-0.25876000	-1.39239200	-1.35392800
H	-3.20070800	1.82116700	2.33442100
H	-0.05383700	-1.13293400	2.28876800

Structure and coordinates of 1′-Int-p4.log

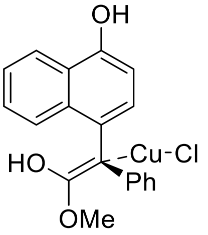

C	-2.61665400	2.97649200	0.96452600
C	-2.22399100	1.70872000	0.54516000
C	-1.07958700	1.53400400	-0.24431600
C	-0.33224700	2.66587200	-0.59096600
C	-0.72304900	3.93646300	-0.17034500
C	-1.86742100	4.09837900	0.60758900
H	-3.50587100	3.08640900	1.57826900
H	-2.80656800	0.84067200	0.84427200
H	0.56592900	2.55481300	-1.19113300
H	-0.12715200	4.79996400	-0.45176200
H	-2.16924800	5.08724900	0.93998200
C	-0.61487800	0.18002100	-0.71863900
C	-1.50370300	-0.59881900	-1.46012200
O	-1.07582000	-1.65366700	-2.17873100

O	-2.74979800	-0.16260000	-1.67344600
C	-3.80316400	-1.12488100	-1.81564200
H	-3.74733100	-1.87709900	-1.02086200
H	-4.73014100	-0.56174600	-1.71644400
H	-3.78082500	-1.59436600	-2.80425400
Cu	-1.22702900	-1.27109800	0.65389800
Cl	-1.88107200	-2.72167600	2.11962300
C	1.41390500	-0.20398800	-2.12672600
C	0.86717800	-0.01308200	-0.87704700
C	1.75392600	0.07013500	0.25258400
C	3.15700800	-0.09864300	0.06145600
C	3.65693900	-0.32462700	-1.25684300
C	2.80056700	-0.36346500	-2.32916100
H	0.24410900	0.48866900	1.76584000
H	0.76644700	-0.23512100	-2.99664100
C	1.30036900	0.32345300	1.57813100
C	4.03828800	-0.04243500	1.17315400
C	3.55766200	0.18827000	2.43866800
C	2.17233600	0.37781900	2.63861900
H	5.10055500	-0.18487800	1.00716600
H	4.23960700	0.22810100	3.28251600
H	1.79108300	0.56893900	3.63719900
O	4.99886700	-0.48612400	-1.37766600
H	5.23085400	-0.62444800	-2.30526200
H	3.18648600	-0.51730900	-3.33348800
H	-1.80275700	-2.24854200	-2.41377500

Structure and coordinates of 1′-Int-o5.log

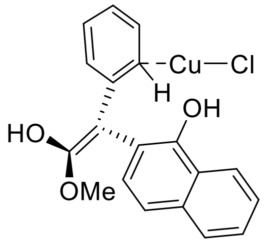

C	1.40043500	-2.70586600	-1.33542000
C	0.53733500	-1.64965200	-1.03577500
C	0.96603300	-0.31152100	-1.04630900
C	2.32759100	-0.07262000	-1.40219300
C	3.19323300	-1.14688000	-1.70038300
C	2.73214300	-2.47343600	-1.65410200
H	1.01708500	-3.72152800	-1.31105300
H	-0.49568400	-1.87542200	-0.79237300
H	2.67103500	0.94050400	-1.57386600
H	4.19712300	-0.93327000	-2.05834700

H	3.40288600	-3.29252700	-1.89056900
C	0.00653500	0.75746900	-0.72706200
C	0.33702600	2.05854000	-0.47028300
O	-0.56276900	3.00916800	-0.18489500
O	1.58821100	2.50154100	-0.46806800
C	1.85842500	3.85539600	-0.07197100
H	2.94019200	3.95739200	-0.14629500
H	1.36967300	4.56153100	-0.74595100
H	1.53794000	4.02779300	0.95770000
H	-1.45749700	2.63425900	-0.22029600
Cu	3.32480800	-0.53122000	0.39896000
Cl	4.02997800	-0.51679100	2.44767100
C	-2.24791100	0.39952800	-1.84469000
C	-3.57944600	0.08145200	-1.80589900
C	-4.20086900	-0.23959200	-0.56599100
C	-3.42700300	-0.24036700	0.62762700
C	-2.03938200	0.08907700	0.54172100
C	-1.44931400	0.41910800	-0.66663900
H	-6.17304700	-0.56526200	-1.39300100
H	-1.76890900	0.64377300	-2.78899500
H	-4.17400700	0.07291000	-2.71500700
C	-5.58152300	-0.56525100	-0.48121300
C	-4.04540600	-0.56224300	1.86422600
C	-5.38430300	-0.87052100	1.91127400
C	-6.15998800	-0.87162200	0.72690300
H	-3.45000500	-0.56065800	2.77115900
H	-5.85252600	-1.11286300	2.86049200
H	-7.21699900	-1.11566800	0.77816400
O	-1.34124200	0.05881300	1.70127300
H	-0.41157900	0.26768300	1.51859200

Structure and coordinates of 1′-Int-p5.log

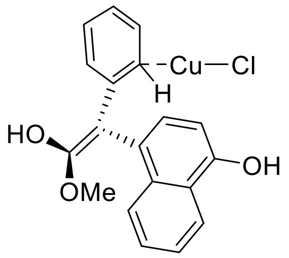

C	-0.98922700	2.58288400	1.17934500
C	-0.26944700	1.42599300	0.86527500
C	-0.61625900	0.60943600	-0.22001000
C	-1.72405600	1.02000800	-1.01512500
C	-2.44939000	2.18626300	-0.69297400

C	-2.08427400	2.96849800	0.41852300
H	-0.68347700	3.18123300	2.03224400
H	0.58003100	1.15658000	1.48406300
H	-1.94422700	0.50201600	-1.94066200
H	-3.22831100	2.53047400	-1.36844600
H	-2.63971200	3.86993500	0.65528300
C	0.20150800	-0.58957200	-0.51794600
C	-0.23557100	-1.63289300	-1.25705300
O	0.53221600	-2.73082400	-1.43939100
O	-1.43896600	-1.67585800	-1.86538300
C	-2.36326000	-2.63791900	-1.32245500
H	-3.32520100	-2.42984700	-1.79038500
H	-2.05710100	-3.66152100	-1.55960000
H	-2.44346100	-2.51256500	-0.23842000
Cu	-3.37055400	0.44974600	0.23651300
Cl	-4.77487400	-0.74697600	1.36468300
C	1.77102600	-1.55275300	1.15279000
C	1.55555300	-0.68601100	0.10665800
C	2.63785000	0.14308700	-0.33409800
C	3.90351300	0.06244100	0.31627000
C	4.06770400	-0.85997900	1.39371800
C	3.02213200	-1.65308200	1.79979000
H	1.54200700	1.13392600	-1.92005000
H	0.95524900	-2.18120500	1.49902600
C	2.49743100	1.06378900	-1.40930100
C	4.97262600	0.89107200	-0.11599100
C	4.79735900	1.77380900	-1.15391200
C	3.54536800	1.85849700	-1.80710900
H	5.93133300	0.81653000	0.38609100
H	5.61886700	2.40659400	-1.47608800
H	3.41353600	2.55750200	-2.62784400
O	5.29463900	-0.89791000	1.97671200
H	5.29584900	-1.54000100	2.69812800
H	0.20424800	-3.22352700	-2.20510200
H	3.15266600	-2.35481500	2.61939400

Structure and coordinates of 1′-Int-o5*.log

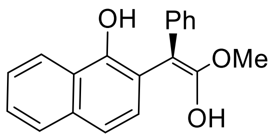

C	4.28403200	-1.61703800	-0.35964300
C	3.35953700	-0.62892100	-0.69120800
C	2.13040700	-0.53623900	-0.02113100

C	1.84657300	-1.48733800	0.97093300
C	2.76711300	-2.47892200	1.29929700
C	3.99427200	-2.54632600	0.63891600
H	5.23045100	-1.66621000	-0.89060600
H	3.59116400	0.08004500	-1.48137200
H	0.89383800	-1.44407300	1.49173800
H	2.52546400	-3.20006200	2.07488400
H	4.71334300	-3.31858100	0.89507900
C	-3.66479000	0.69410300	1.11742500
C	-2.63407100	0.16010800	0.29533600
C	-4.95790200	0.24051300	1.01742800
C	-1.27218600	0.59753400	0.37187900
C	-2.97086100	-0.86119600	-0.64029500
C	-5.29094200	-0.77208700	0.08795900
H	-5.72815000	0.66285700	1.65526800
C	-0.29949800	0.06390700	-0.45540900
C	-1.95820800	-1.41148700	-1.46961000
C	-4.31816400	-1.30620900	-0.71977300
H	-6.31595700	-1.12268600	0.01541600
C	-0.67046000	-0.95720300	-1.36961100
H	-2.22033200	-2.18907900	-2.18104200
H	-4.56443800	-2.08383000	-1.43792700
H	0.10341100	-1.37750900	-2.00612600
H	-3.45814500	1.47714100	1.84209200
C	1.12305600	0.49484100	-0.37161700
C	1.44229600	1.77774800	-0.64463900
O	0.55132000	2.60895700	-1.22132000
O	2.62244100	2.40118900	-0.44315600
C	3.30500100	2.21045000	0.80263600
H	3.79615500	3.15912500	1.02447800
H	2.59300100	1.96240400	1.59364000
H	4.05492900	1.42102400	0.71868600
H	0.84560400	3.51943000	-1.07814400
O	-0.88953700	1.55075100	1.26257500
H	-1.62535600	1.81539900	1.82591700

Structure and coordinates of 1′-Int-p5*.log

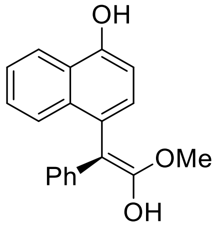

C	-2.26159300	2.14450700	1.92762000

C	-1.52456700	1.03132800	1.52787800
C	-1.76404100	0.41458200	0.29177800
C	-2.74929100	0.96626600	-0.54174200
C	-3.48913900	2.07545000	-0.14210000
C	-3.25045600	2.67040100	1.09690400
H	-2.06124400	2.60356200	2.89149500
H	-0.75311600	0.63537600	2.18268300
H	-2.93009700	0.52295600	-1.51688500
H	-4.24577200	2.48245300	-0.80687000
H	-3.82223700	3.53992000	1.40705400
C	-0.94196800	-0.74388400	-0.14403500
C	-1.45777600	-1.78552300	-0.83068400
O	-2.76167100	-2.06492500	-1.05340800
C	-3.66608200	-2.06763300	0.05951500
H	-4.42979700	-2.80977500	-0.17666500
H	-3.14220800	-2.34962400	0.97691200
H	-4.13375700	-1.08872600	0.18606500
C	1.02511000	-1.66763200	1.06622800
C	0.51394600	-0.74181300	0.18517400
C	1.40553600	0.23097500	-0.37672700
C	2.78258900	0.23038400	-0.00904200
C	3.25231100	-0.75832000	0.90671300
C	2.39024300	-1.69155000	1.42808100
H	-0.07229800	1.21400400	-1.62043400
H	0.35855900	-2.40790800	1.49999000
C	0.96869000	1.20608200	-1.31570300
C	3.66219800	1.19758300	-0.56320600
C	3.20018100	2.13147700	-1.45792500
C	1.83874400	2.13072700	-1.84011800
H	4.70711600	1.18587200	-0.27226600
H	3.88006500	2.86775800	-1.87586400
H	1.48007000	2.86487600	-2.55547200
O	4.57576400	-0.71580800	1.21755300
H	4.79101300	-1.42908100	1.83237000
O	-0.66151000	-2.69175700	-1.43868900
H	-1.20756200	-3.44918800	-1.69242000
H	2.75280300	-2.44299600	2.12500700

Structure and coordinates of 1′-TS-o6-2w.log

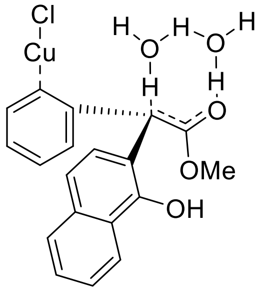

C	3.49192200	-0.53127100	-1.61497100
C	2.62381700	0.30323800	-0.88099800
C	1.21936300	0.31928700	-1.14306200
C	0.75961200	-0.52674500	-2.16531200
C	1.62694200	-1.34383500	-2.89557800
C	2.99029000	-1.36533800	-2.62698100
H	4.56228400	-0.47100900	-1.43770000
H	3.04489100	1.03017500	-0.19210200
H	-0.30022500	-0.54444000	-2.39942000
H	1.22119100	-1.97300300	-3.68241600
H	3.66091600	-2.00331000	-3.19270900
C	0.28727800	1.13631100	-0.32786800
C	0.59377600	2.50293200	-0.03190700
O	-0.10927700	3.26901100	0.67358800
O	1.74834500	2.97584400	-0.53530600
C	2.15602000	4.28218400	-0.12304300
H	3.12092900	4.44741500	-0.60284500
H	1.43988600	5.03705600	-0.45500700
H	2.26600500	4.33355100	0.96345400
O	-1.07803700	2.21365900	2.75972900
H	-0.98630300	2.79742300	3.52567600
H	-0.83935600	2.73308400	1.91541500
O	0.50461200	0.47546200	2.33805200
H	0.61002400	0.68893000	1.31924900
H	-0.23356700	1.29592000	2.70280500
H	1.36827000	0.45120000	2.77629100
C	-3.51344400	-2.08061800	0.30537800
C	-3.03201600	-0.80171200	-0.08386000
C	-4.85377000	-2.37753200	0.22196200
C	-1.64345500	-0.46586000	-0.00118400
C	-3.95248800	0.16876300	-0.56327800
C	-5.77284000	-1.41304100	-0.25602500
H	-5.21100800	-3.35782200	0.52385700
C	-1.16989900	0.78418800	-0.37735700
C	-3.46189700	1.44184200	-0.96257900
C	-5.32961900	-0.16973100	-0.63977400

H	-6.82888800	-1.65875700	-0.32056500
C	-2.12567700	1.72080900	-0.87382700
H	-4.15707000	2.18290400	-1.34768500
H	-6.02950300	0.57561600	-1.00905200
H	-1.76539700	2.69260600	-1.19641000
H	-2.80990000	-2.82067500	0.67080600
Cu	2.69042700	-1.36314500	0.33915700
Cl	2.51038100	-2.58088200	2.11268100
O	-0.84990900	-1.47440800	0.44461100
H	0.07860200	-1.21044500	0.48340900

Structure and coordinates of 1′-TS-p6-2w.log

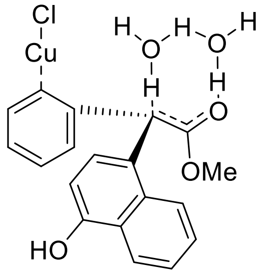

C	-3.32054300	0.47221900	1.43683000
C	-2.25782100	0.96983000	0.65444600
C	-0.90102200	0.75091200	1.02968500
C	-0.67895100	-0.00033000	2.19017900
C	-1.73283300	-0.51296100	2.95505800
C	-3.05340100	-0.28822700	2.59243800
H	-4.34362800	0.75256200	1.19639800
H	-2.47793500	1.63405300	-0.17657900
H	0.34088200	-0.18787400	2.51152000
H	-1.50748200	-1.08571400	3.85005500
H	-3.87173300	-0.67211100	3.19250500
C	0.22687100	1.25755300	0.20569300
C	0.25342600	2.64001000	-0.15458900
O	1.13833000	3.20927600	-0.84703600
O	-0.79703100	3.37688900	0.25766500
C	-0.91120500	4.70318200	-0.25882500
H	-1.83748800	5.09777700	0.16018700
H	-0.06788000	5.32226500	0.05621000
H	-0.96973500	4.69450500	-1.35086100
O	2.07847800	1.80948000	-2.70909600
H	2.25520200	2.33770700	-3.50004300
H	1.86712400	2.43632700	-1.92351900
O	0.01085600	0.63282600	-2.47865200
H	-0.08409100	0.82827100	-1.45034000
H	1.01808600	1.17737000	-2.74382500

H	-0.75239700	1.00844700	-2.94324200
Cu	-2.92695600	-0.88401400	-0.19221300
Cl	-3.20164300	-2.41900400	-1.69306900
C	1.55642900	0.57734900	0.37323100
C	1.73500900	-0.82181800	0.07700400
C	2.65078900	1.26892100	0.85243100
C	0.67585300	-1.64947800	-0.39207800
C	3.00741900	-1.44096600	0.26959100
C	3.91932700	0.67580500	1.02554300
H	2.54681100	2.31892600	1.10599400
C	0.85943700	-2.98543900	-0.65071500
H	-0.30813500	-1.22103000	-0.54538200
C	3.17597900	-2.82435600	-0.00670800
C	4.09955500	-0.65414200	0.74011200
H	4.74796500	1.27396200	1.39692800
C	2.12455000	-3.58492000	-0.45543200
H	0.02559600	-3.58252000	-1.00817900
H	4.15187000	-3.27297700	0.14550500
H	2.26265700	-4.64230000	-0.66031600
O	5.29247100	-1.29197300	0.89265300
H	5.95591900	-0.66902600	1.21571000

Structure and coordinates of 1′-Int-p7.log

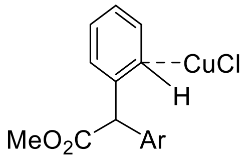

C	-3.11313700	1.47704500	0.57920900
C	-2.00588600	1.42987200	-0.29168300
C	-0.70065800	1.24981600	0.22221800
C	-0.52989500	1.11864400	1.59392500
C	-1.62953300	1.16198900	2.46160400
C	-2.91413700	1.33491500	1.96695700
H	-4.09960200	1.71655100	0.19106800
H	-2.13887400	1.64124700	-1.35130500
H	0.46719400	0.98079500	2.00083600
H	-1.47029100	1.05544300	3.52997000
H	-3.76438500	1.37321800	2.63988200
C	0.47075000	1.24094100	-0.75200200
Cu	-2.87778000	-0.54243500	-0.17914800
Cl	-3.52714700	-2.58158600	-0.52066800
C	1.65421100	0.39964700	-0.28885400
C	1.55171400	-1.03191000	-0.31870300
C	2.81616400	0.96786300	0.17442400

C	0.37994800	-1.71095500	-0.75326100
C	2.65310800	-1.82803200	0.11340300
C	3.91484900	0.19070600	0.60569500
H	2.92059700	2.04739100	0.22523500
C	0.30838200	-3.08305400	-0.76517900
H	-0.49181800	-1.14490700	-1.06894200
C	2.55916200	-3.24377800	0.08438400
C	3.83949100	-1.17871900	0.57393000
H	4.81544200	0.68415300	0.96029400
C	1.41051900	-3.86123500	-0.34770500
H	-0.60744300	-3.56690400	-1.09035000
H	3.40876000	-3.83309800	0.41216600
H	1.34453300	-4.94485800	-0.36349900
O	4.85637700	-1.98628300	0.97024300
H	5.60808800	-1.45226900	1.25847100
H	0.11233900	0.83114400	-1.70124900
C	0.90979100	2.65562100	-1.12064600
O	1.27628600	2.97297800	-2.23343000
O	0.86122900	3.49859900	-0.09106000
C	1.28992800	4.84594300	-0.33950600
H	2.33204300	4.86031600	-0.66695200
H	0.65658300	5.31204700	-1.09739800
H	1.18419300	5.36303100	0.61312300

Structure and coordinates of Pro-p8.log

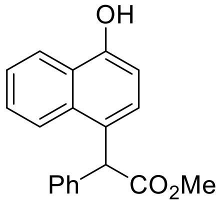

C	-4.31193600	-1.92065500	-0.21088100
C	-3.42030700	-1.02664600	-0.79440600
C	-2.14758000	-0.82038700	-0.24813700
C	-1.78287200	-1.53494300	0.89272900
C	-2.67558700	-2.43454600	1.47948200
C	-3.94095700	-2.63013500	0.93268100
H	-5.29386800	-2.06712700	-0.65108200
H	-3.71350100	-0.47979400	-1.68811600
H	-0.80024200	-1.39866400	1.33312300
H	-2.37539900	-2.98330500	2.36735300
H	-4.63289800	-3.33140000	1.38940900
C	-1.22224000	0.16761400	-0.95284900
C	-1.76923500	1.59289800	-0.93125400

O	-1.67920000	2.36006900	-1.86819600
O	-2.32881200	1.91598400	0.23399500
C	-2.82256100	3.25700500	0.36142100
H	-3.23822300	3.32045800	1.36601100
H	-2.00865000	3.97573000	0.24188900
H	-3.59921000	3.44807600	-0.38230000
H	-1.20438800	-0.09896300	-2.01335900
C	3.52334900	-1.22216400	-1.51198800
C	2.52792400	-0.53402300	-0.76531300
C	4.82912800	-1.25238400	-1.09116100
C	1.17765000	-0.49768400	-1.20159800
C	2.90877000	0.12944900	0.43722200
C	5.20526700	-0.59172100	0.10220600
H	5.57770500	-1.78275000	-1.67174700
C	0.22134100	0.16485500	-0.47065700
C	1.88808700	0.81323300	1.16963800
C	4.26857100	0.08133900	0.84811500
H	6.23962300	-0.61868200	0.43031700
C	0.58733100	0.82528900	0.72763700
H	4.59745100	0.57278400	1.76015300
H	-0.15669700	1.34615400	1.32149500
H	3.22788500	-1.72678700	-2.42776700
H	0.91119600	-1.00502800	-2.12495000
O	2.14799600	1.47325200	2.32873900
H	3.08448900	1.43649500	2.55594000

Structure and coordinates of React-CuCl2.log

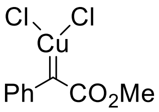

C	2.45940900	-2.62337700	0.04199700
C	1.34339400	-1.82192600	-0.08180000
C	1.47577300	-0.40692700	-0.18658900
C	2.78029800	0.16973300	-0.16496700
C	3.88984500	-0.64040900	-0.04195000
C	3.72690500	-2.02907800	0.06434000
H	2.35814800	-3.69952600	0.12503300
H	0.34474000	-2.24917300	-0.10957400
H	2.90247200	1.24545300	-0.24519500
H	4.88394600	-0.20824500	-0.02315500
H	4.60630400	-2.65812100	0.16509300
C	0.31234400	0.36486300	-0.30172400
C	0.40876000	1.82202800	-0.55379200
O	0.42430300	2.21500200	-1.70540800

O	0.42510300	2.57241800	0.52810500
C	0.43736500	4.00145600	0.32187500
H	0.42693200	4.43312800	1.32045200
H	1.34213200	4.29568500	-0.21325600
H	-0.45001000	4.30646200	-0.23573100
Cu	-1.47393400	-0.38922000	-0.19934400
Cl	-3.34899600	-1.31367500	-0.88231300
Cl	-1.44337900	-0.18607900	2.04886600

Structure and coordinates of 2-TS-p1.log

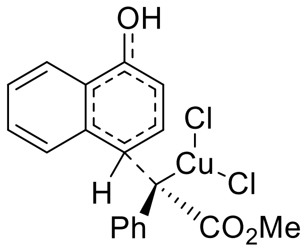

C	1.56072000	-1.52753800	2.40707700
C	0.50963600	-1.16743400	1.57169700
C	0.33100500	0.17895700	1.16906300
C	1.25790600	1.14915000	1.63831800
C	2.30155100	0.78330400	2.46396900
C	2.45957900	-0.55809600	2.84352900
H	1.68133900	-2.56310500	2.70706400
H	-0.19390400	-1.92213100	1.23533800
H	1.15261100	2.18780700	1.34079100
H	3.00577900	1.53152200	2.81258200
H	3.28513000	-0.83950000	3.49053800
C	-0.72873300	0.52449300	0.27215700
C	-1.03604700	1.98184000	0.08542600
O	-0.79687100	2.68893400	-0.86918500
O	-1.68435400	2.39372200	1.17743200
C	-2.20118600	3.73445900	1.15082200
H	-2.69200100	3.87128900	2.11335100
H	-1.38902900	4.45513900	1.03283400
H	-2.92236500	3.84262000	0.33791800
Cu	-2.27476900	-0.68215000	0.03436900
Cl	-3.11404400	-2.68580100	0.53965300
Cl	-3.67509200	0.57123600	-1.20207800
C	0.86825300	-2.21652700	-1.41942100
C	2.13662800	-1.89279800	-0.95076300
C	2.62870800	-0.54233200	-0.99889500
C	1.75063700	0.46901100	-1.47702200
C	0.40433900	0.13346400	-1.83125300

C	0.02725800	-1.21187300	-1.88076600
H	4.63897700	-0.93751400	-0.22787800
H	0.55130100	-3.25351700	-1.40604000
C	3.94015500	-0.19219000	-0.59784500
C	2.21634400	1.80070400	-1.53872300
H	-0.22424400	0.88980700	-2.28573700
H	-0.95794000	-1.47314500	-2.25683100
C	3.49993500	2.11756400	-1.14809300
C	4.36793200	1.11516400	-0.67373900
H	1.53830300	2.57059400	-1.89128200
H	3.84564700	3.14498800	-1.20713800
H	5.37713300	1.37159800	-0.36805500
O	2.87948900	-2.90849800	-0.49872300
H	3.73304500	-2.61569700	-0.15237100

Structure and coordinates of 2-TS-o1.log

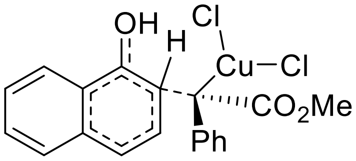

C	-1.91052100	0.55617700	-2.47582100
C	-0.88292600	0.12243900	-1.65732400
C	-0.19944200	1.02291900	-0.80240600
C	-0.63156400	2.37311500	-0.77733200
C	-1.66747700	2.80247000	-1.59287800
C	-2.30669600	1.89611800	-2.44099000
H	-2.41244900	-0.14583400	-3.13321400
H	-0.58354000	-0.92140500	-1.68334900
H	-0.14192800	3.08322500	-0.11894200
H	-1.98233900	3.84047300	-1.56584200
H	-3.11636700	2.23495700	-3.08037700
C	0.88533200	0.56241900	0.01846700
C	1.85361500	1.59182800	0.54252400
O	2.15570800	1.81439000	1.69486900
O	2.40454500	2.20956100	-0.50388700
C	3.49255600	3.10144400	-0.21572000
H	3.83661500	3.45923400	-1.18513900
H	3.14510800	3.93661000	0.39680100
H	4.29446700	2.56799700	0.29918800
Cu	1.70186300	-1.21102900	-0.36302300
Cl	1.02240000	-3.26042400	-1.01778000
Cl	3.87555200	-0.81927800	-0.09265400
C	-0.11304300	0.13347700	2.13876300

C	-0.79536700	-0.98174100	1.63650700
C	-2.09303800	-0.84021700	1.06212900
C	-2.71589200	0.43950500	1.14677900
C	-2.06615200	1.51840300	1.83373600
C	-0.81027400	1.36029400	2.33271000
H	-2.29954000	-2.87802100	0.30561000
H	0.84714300	-0.03048900	2.61362200
C	-2.75332900	-1.89449200	0.38428900
C	-3.97972300	0.61831800	0.54313900
H	-2.59479900	2.46094400	1.93757500
H	-0.29723300	2.17650700	2.82810200
C	-4.59544000	-0.41886600	-0.12427500
C	-3.97950700	-1.68470700	-0.20464600
H	-4.45615000	1.59219900	0.60523200
H	-5.56258000	-0.26352000	-0.59261300
H	-4.47250100	-2.49520900	-0.73123600
O	-0.15113400	-2.14824200	1.72324100
H	-0.48274900	-2.82197800	1.10993600

Structure and coordinates of 2-Int-p2.log

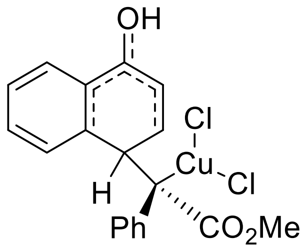

C	0.77751900	-2.15286100	2.46265000
C	0.11779500	-1.55909300	1.38722500
C	0.14734400	-0.17230100	1.19058800
C	0.87157400	0.60473400	2.10960500
C	1.53500700	0.01599000	3.18125600
C	1.49141600	-1.36710500	3.36360200
H	0.72469300	-3.22933200	2.59613200
H	-0.43718000	-2.18949800	0.69816500
H	0.92971900	1.68081900	1.98010800
H	2.09207900	0.64026600	3.87385700
H	2.00527200	-1.82535700	4.20338200
C	-0.93835500	1.86158000	0.09190100
O	-0.84587900	2.72222400	-0.76770500
O	-1.54397700	2.08788300	1.26735900
C	-2.12006100	3.38545200	1.45180300
H	-2.58123200	3.35841000	2.43839000
H	-1.34781000	4.15780100	1.41686700

H	-2.87533600	3.58206200	0.68716100
C	-0.47698600	0.44433300	-0.03975100
Cu	-2.29370400	-0.45435700	-0.28888000
C	4.19973500	0.05638300	-0.52730100
C	2.89036300	-0.29739100	-0.91565000
C	4.49920200	1.37651100	-0.25991000
C	2.57990800	-1.65169500	-1.27332900
C	1.87011700	0.67456600	-1.01338100
C	3.50166500	2.35124300	-0.39707000
H	5.50336500	1.65923700	0.03736400
C	1.30978900	-2.02925700	-1.78467000
C	0.44835600	0.27121100	-1.30717000
C	2.20767400	2.00744200	-0.76498200
H	3.74025100	3.39407600	-0.21139300
C	0.31513600	-1.11331200	-1.81194000
H	1.15450300	-3.05019000	-2.11945700
H	0.01749300	0.94591500	-2.05951200
H	1.44811600	2.77443700	-0.85715500
H	-0.66183200	-1.40341700	-2.18856800
H	4.96543600	-0.70769500	-0.45350600
O	3.53627000	-2.53142700	-1.14655000
H	3.25534400	-3.42106000	-1.42353300
Cl	-2.91605700	0.24690200	-2.33120200
Cl	-3.66923400	-1.87385100	0.80611600

Structure and coordinates of 2-Int-o2.log

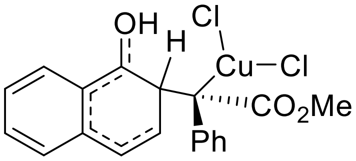

C	-0.97258300	2.05049000	-2.36777000
C	-0.23330100	1.10370100	-1.66212700
C	0.02502700	1.25875900	-0.29444300
C	-0.48080500	2.39799400	0.34732300
C	-1.21896100	3.34814000	-0.35496600
C	-1.47061500	3.17674800	-1.71594300
H	-1.15105400	1.90809400	-3.42951700
H	0.16134300	0.23915600	-2.18723400
H	-0.30056400	2.54494300	1.40818300
H	-1.59918100	4.22351700	0.16339900
H	-2.04447900	3.91786200	-2.26440200
C	1.67327200	0.60119500	1.55572700
O	1.90581800	-0.02328200	2.58029100

O	2.31233000	1.73310000	1.23937400
C	3.36288300	2.13974600	2.12232500
H	3.76034800	3.06022200	1.69594300
H	2.97318000	2.32341500	3.12647300
H	4.14344800	1.37598500	2.16224900
C	0.71446200	0.16669300	0.49574800
Cu	2.11416700	-0.85727000	-0.61990900
C	-3.45606500	-1.38069900	-1.11193300
C	-2.64051100	-1.03443500	-0.00386800
C	-4.79459500	-1.06445500	-1.11131100
C	-1.25024200	-1.31941600	0.02771600
C	-3.20805800	-0.35458800	1.11166100
C	-5.35486100	-0.40354900	-0.00297100
H	-5.41485900	-1.32147900	-1.96264800
C	-0.34459900	-0.83365400	1.10430300
C	-2.38977400	0.01015900	2.25053200
C	-4.57750700	-0.05487300	1.08768000
H	-6.41307200	-0.16130800	-0.00609000
C	-1.06945800	-0.21410800	2.25424900
H	0.22000900	-1.70595100	1.46591200
H	-2.87962000	0.47963300	3.09780700
H	-5.02151100	0.45631600	1.93572000
H	-0.45930400	0.06837300	3.10391700
H	-3.04928700	-1.87661900	-1.98891000
Cl	2.32535900	-2.92075500	0.17777100
Cl	3.23807100	0.35938800	-2.09215200
O	-0.65042600	-2.00602300	-0.89335100
H	-1.23855400	-2.32155100	-1.60104600

Structure and coordinates of 2-TS-p3.log

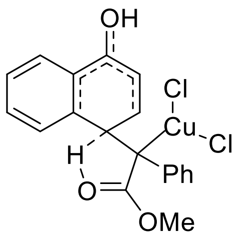

C	0.51420600	3.04709700	-1.59957900
C	0.13953400	1.80700400	-1.10586000
C	0.69689300	1.29742300	0.09462400
C	1.64162300	2.10489900	0.77658600
C	2.00689700	3.34527400	0.27125500
C	1.45205900	3.82365700	-0.91643900
H	0.07611200	3.40902600	-2.52447400
H	-0.58379400	1.21355200	-1.65647300

H	2.09250700	1.75019700	1.69405800
H	2.73696100	3.94208000	0.80915800
H	1.74929500	4.79139400	-1.30816400
C	0.54838000	-0.62043000	1.86733500
O	-0.20261600	-1.55706200	2.23149300
O	1.52568000	-0.18220600	2.61650700
C	1.77806000	-0.87214900	3.85860300
H	2.64474900	-0.37282700	4.28780900
H	0.91501400	-0.77976600	4.51995500
H	1.99919900	-1.92189700	3.65924600
C	0.30521300	-0.05298400	0.53858000
Cu	1.95191800	-0.56415200	-0.58949300
H	-0.95726300	-1.46890500	1.08410600
C	-1.01832500	-0.65230800	0.07118600
C	-2.25865600	0.08677900	0.34526700
C	-1.04580200	-1.59276000	-0.99476100
C	-2.32576200	1.09587100	1.32815900
C	-3.44434600	-0.24456300	-0.35582700
C	-2.20294300	-1.93117200	-1.66725600
H	-0.12248200	-2.10782100	-1.24459600
C	-3.51395100	1.74534000	1.59099900
H	-1.43307500	1.37395100	1.87916800
C	-4.65626200	0.42885300	-0.07109300
C	-3.38846800	-1.26531100	-1.36119200
H	-2.19089700	-2.70004900	-2.43324200
C	-4.68927900	1.41286900	0.88953000
H	-3.54142700	2.52353800	2.34750500
H	-5.55546600	0.16087400	-0.61459900
H	-5.61719300	1.93107000	1.10791200
O	-4.52763700	-1.54313000	-1.98320700
H	-4.40255800	-2.24292900	-2.64287100
Cl	3.46992600	0.10193100	-2.10084400
Cl	2.29400600	-2.72912000	-0.02552500

Structure and coordinates of 2-TS-o3.log

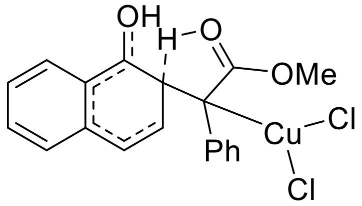

C	-1.02227700	3.62898600	-0.01262500
C	-0.47102700	2.36931200	-0.20409700
C	-1.26037900	1.28800700	-0.65706700

C	-2.63278800	1.53098900	-0.89606600
C	-3.17477700	2.79574300	-0.69993000
C	-2.37756200	3.85159200	-0.25855100
H	-0.39017900	4.43943500	0.33724600
H	0.58306300	2.22046600	0.00834500
H	-3.27501300	0.72491400	-1.22660900
H	-4.23235800	2.95383800	-0.88771500
H	-2.80949000	4.83523400	-0.10291700
C	-1.13805600	-1.13037600	-1.65257700
O	-0.30913900	-1.99442100	-2.02001900
O	-2.39305800	-1.16896900	-2.01251000
C	-2.83125700	-2.31107100	-2.77492100
H	-3.89117700	-2.14106400	-2.95456700
H	-2.28601500	-2.36484200	-3.71889600
H	-2.68213700	-3.22225300	-2.19351200
C	-0.64821800	-0.05612900	-0.77503100
C	3.91593500	-0.62802400	1.35898300
C	3.09243400	-0.24545100	0.26947200
C	5.27164500	-0.40114500	1.31700900
C	1.67529400	-0.43573600	0.27624000
C	3.67514600	0.38379600	-0.86649800
C	5.85028300	0.21841400	0.19077000
H	5.89680600	-0.70156500	2.15097400
C	0.87567300	-0.14973200	-0.86916800
C	2.85804800	0.79677700	-1.97463400
C	5.06926100	0.60212800	-0.87773400
H	6.92118200	0.39455700	0.16756900
C	1.52745400	0.56965300	-1.96587200
H	0.76438500	-1.30103200	-1.40939200
H	3.33385600	1.29389800	-2.81390200
H	5.51674200	1.07924900	-1.74412900
H	0.90273700	0.86403900	-2.80380100
H	3.50830400	-1.11397100	2.24116100
Cu	-1.41904200	-0.47492400	1.08615700
Cl	-1.94957600	-2.67050700	1.03569300
Cl	-1.84187900	0.52591900	3.05408300
O	1.02985900	-0.91671600	1.31881800
H	1.59596800	-1.15565900	2.06887200

Structure and coordinates of 2-Int-p4.log

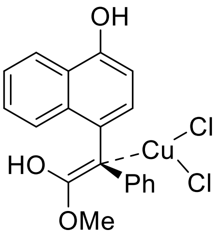

C	1.36939600	3.17519200	-1.32459600
C	0.62814600	2.17130600	-0.72036700
C	1.18280400	1.35659700	0.30131900
C	2.52779300	1.61053200	0.67922900
C	3.25734700	2.61940500	0.06430600
C	2.68857400	3.40553600	-0.93753200
H	0.91572200	3.77282300	-2.10856700
H	-0.39340500	2.00438600	-1.04206100
H	2.99985300	1.02001100	1.45133800
H	4.28447900	2.78912800	0.37136800
H	3.27006700	4.18834200	-1.41416400
C	0.32843500	0.26866400	0.85712000
C	0.70104500	-0.53289700	1.96952900
O	-0.14717300	-1.33931700	2.55668000
O	1.89250500	-0.52518000	2.47582000
C	2.25280600	-1.49080000	3.49385100
H	3.30640100	-1.30152900	3.68657200
H	1.65971800	-1.32094600	4.39317400
H	2.10732700	-2.49915800	3.10573000
Cu	1.42339100	-0.64572500	-0.75422700
Cl	2.22410900	-0.41137100	-2.81853300
Cl	1.80553400	-2.75682500	-0.04866400
C	-3.04183300	1.89282300	1.38852300
C	-3.90328600	1.20169800	0.56861700
C	-3.43921400	0.08715500	-0.19721500
C	-2.06350200	-0.30482600	-0.09394600
C	-1.17211000	0.46406200	0.72766400
C	-1.69064800	1.52375900	1.44943900
H	-5.35030600	-0.35354500	-1.17003400
C	-4.30949700	-0.64359400	-1.05229800
C	-1.66205200	-1.46594000	-0.81305300
H	-1.02447200	2.11360200	2.07335600
C	-2.52824200	-2.15662100	-1.62207500
C	-3.86773600	-1.73326900	-1.75872300
H	-0.65589200	-1.85186500	-0.69911900
H	-2.17922100	-3.03747300	-2.15172900
H	-4.54763600	-2.27566700	-2.40770200
O	-5.18800000	1.62511200	0.53696300

H	-5.73464500	1.08231500	-0.04351400
H	-1.03587400	-1.25332500	2.16642300
H	-3.41240500	2.73063800	1.96941000

Structure and coordinates of 2-Int-o4.log

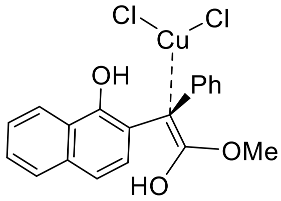

C	1.81152400	3.89007700	0.27205100
C	1.26319300	2.70016000	0.74407000
C	1.59529200	1.47935100	0.13951200
C	2.49137400	1.47033300	-0.93650700
C	3.03645400	2.66249400	-1.40730000
C	2.69846300	3.87344700	-0.80456200
H	1.54589900	4.82998600	0.74600300
H	0.56430700	2.71437600	1.57614200
H	2.75155800	0.52553300	-1.40441000
H	3.72602200	2.64266700	-2.24565300
H	3.12189300	4.80180200	-1.17569000
C	0.99283300	0.20997400	0.67878200
C	1.89663600	-0.62610400	1.37326400
Cu	0.39983600	-1.29837000	-0.74418800
Cl	2.35790300	-2.25026500	-1.34757600
Cl	-1.30948000	-2.20817100	-1.85642200
C	-0.47456200	0.11373300	0.83820600
C	-1.34437600	0.74883000	-0.08447000
C	-2.76738700	0.66993500	0.06194400
C	-3.31800600	-0.02351500	1.16661300
C	-2.43552100	-0.61753700	2.12107600
C	-1.08942100	-0.54908100	1.96453800
H	-3.20842300	1.79440400	-1.73581800
C	-3.63100500	1.27253900	-0.88528300
C	-4.72237100	-0.09348600	1.30346800
H	-2.85790500	-1.11596800	2.98826600
H	-0.45136600	-0.98982600	2.71700800
C	-5.54729400	0.49967800	0.37205700
C	-4.99670800	1.18534800	-0.73099400
H	-5.14102400	-0.62272300	2.15473900
H	-6.62536400	0.43728800	0.48479900
H	-5.65395900	1.64373500	-1.46321900
O	-0.94482300	1.43789900	-1.16097300
H	0.01188700	1.59760700	-1.16856500

O	1.48872600	-1.71034800	1.99218900
H	2.20627100	-2.30268500	2.27040500
O	3.15587300	-0.29299800	1.37556500
C	4.16069900	-1.17880800	1.90820500
H	5.10384500	-0.66305600	1.74256100
H	4.00842400	-1.32364100	2.98128800
H	4.16239700	-2.12400400	1.35926300

Structure and coordinates of 2-Int-p5*.log

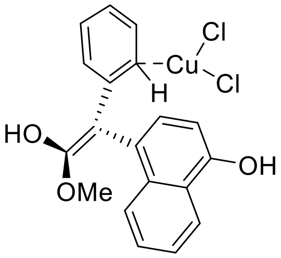

C	-2.84320800	-0.05073200	1.96961000
C	-1.97496800	0.77866700	1.25654700
C	-0.55640400	0.57948200	1.34000200
C	-0.08905900	-0.45206600	2.19370200
C	-0.96482900	-1.25667300	2.90584100
C	-2.34267600	-1.07622000	2.78521000
H	-3.91161400	0.13699200	1.92918600
H	-2.38173500	1.63329100	0.73614900
H	0.97849800	-0.60862400	2.29991500
H	-0.57262900	-2.03243700	3.55495800
H	-3.02880600	-1.71689900	3.32910200
C	0.39971400	1.29775000	0.54510500
C	1.08866700	-1.28082700	-0.79455700
C	2.08677500	-0.45968500	-0.20527400
C	1.40603700	-2.49364900	-1.35457200
C	1.79077400	0.81079400	0.39583000
C	3.43569000	-0.91300900	-0.24534600
C	2.74400700	-2.95046400	-1.36515000
H	0.62195500	-3.09965600	-1.79831700
C	2.82788800	1.59131600	0.86599700
C	4.46421900	-0.07601300	0.28546800
C	3.73955600	-2.17161100	-0.82794400
H	2.98316200	-3.91113300	-1.81057400
C	4.16511000	1.15656700	0.82180900
H	4.77127600	-2.50548900	-0.84924700
H	4.95368100	1.79014200	1.21822300
H	0.05985700	-0.93661900	-0.81998200
Cu	-2.53871900	-0.62361400	-0.40441800

Cl	-3.19287500	-2.76301400	-0.59228400
Cl	-2.01286100	0.83643400	-2.09168300
H	2.61259400	2.55804200	1.31342300
O	5.72509100	-0.56083300	0.22118100
H	6.34877800	0.08064800	0.58694800
C	0.06525500	2.47806200	-0.18281800
O	0.86996900	2.84338700	-1.14343600
H	0.60035000	3.65635300	-1.60357200
O	-0.97124500	3.18186900	0.14430500
C	-1.46041700	4.24997500	-0.70034200
H	-2.39854600	4.55620100	-0.24387100
H	-0.75673500	5.08642400	-0.69146500
H	-1.64302700	3.86409300	-1.70640000

Structure and coordinates of 2-Int-o5*.log

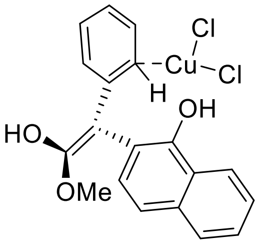

C	2.96785300	-0.96196500	-1.71402400
C	2.05157200	0.06406000	-1.42482500
C	0.67298400	-0.25074000	-1.13729200
C	0.32405500	-1.63652800	-1.09643200
C	1.25092700	-2.63228000	-1.32917900
C	2.57994000	-2.30945600	-1.63370800
H	3.96523300	-0.70635300	-2.05985100
H	2.34390500	1.08576100	-1.61459500
H	-0.69649600	-1.91212000	-0.85769200
H	0.94898800	-3.67225200	-1.26554500
H	3.30857900	-3.09178500	-1.81592700
C	-4.35002500	-0.44331200	1.91661600
C	-3.71004000	-0.29310000	0.65561200
C	-5.66118200	-0.84642000	1.99697900
C	-2.34717400	0.12435400	0.52082800
C	-4.45214400	-0.57101400	-0.52928500
C	-6.39864400	-1.12209800	0.82128600
H	-6.13419500	-0.95500300	2.96795300
C	-1.76741900	0.26416400	-0.72511500
C	-3.83438300	-0.42689600	-1.80048600
C	-5.80621900	-0.98670900	-0.40937600
H	-7.43425900	-1.43914900	0.89817900
C	-2.52941500	-0.02049000	-1.88730500

H	-4.40827400	-0.64282500	-2.69678400
H	-6.36524500	-1.19571500	-1.31760200
H	-2.05257600	0.08990200	-2.85715600
H	-3.82163200	-0.24285400	2.84526800
Cu	3.13168800	-0.38916800	0.39691900
Cl	4.62785200	-1.80661200	1.35683100
Cl	2.54832400	1.34028600	1.72789100
C	-0.34784200	0.70466600	-0.86916000
C	-0.11726800	2.08568000	-0.70692700
O	-1.12810600	2.82946800	-0.31608100
O	1.04272200	2.61815100	-0.95629700
C	1.35871300	3.96846000	-0.56236100
H	2.42288000	4.07607000	-0.75871700
H	0.80139700	4.68159600	-1.17660400
H	1.17000500	4.10284100	0.50495900
H	-0.91533700	3.77437800	-0.24903800
O	-1.56691400	0.39498800	1.59268100
H	-2.03425100	0.25779400	2.42474700

Structure and coordinates of 2-TS-o6-2w.log

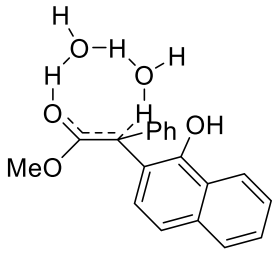

C	-3.69605700	2.46645800	0.58745200
C	-3.04913600	1.23571300	0.64889300
C	-1.83884900	1.02167900	-0.02500800
C	-1.28288200	2.08393500	-0.74419100
C	-1.92479300	3.32090300	-0.80087500
C	-3.13513200	3.51716900	-0.13989300
H	-4.63520800	2.60843800	1.11446900
H	-3.48949700	0.42722000	1.22733000
H	-0.33649500	1.94967300	-1.25905500
H	-1.47305800	4.13300900	-1.36321700
H	-3.63392900	4.48069900	-0.18426400
C	-1.14610200	-0.31748400	0.07894100
C	-1.85685200	-1.44252500	-0.46531400
O	-1.44220600	-2.66063800	-0.38130100
O	-3.01769800	-1.26408000	-1.02570300
C	-3.81932400	-2.41340000	-1.37783600
H	-4.75031600	-1.99505300	-1.75458200

H	-3.31987900	-2.99456000	-2.15392600
H	-4.00193600	-3.02661400	-0.49426100
O	0.16513700	-3.04552000	1.50043700
H	0.08713400	-3.92137000	1.89830800
H	-0.68798800	-2.81569700	0.31868100
O	-0.92597200	-0.98731600	2.58194000
H	-1.21570700	-0.65798000	1.35469400
H	-0.22031300	-2.37611800	2.14074800
H	-1.64071100	-0.81998900	3.20513300
C	3.64087500	0.78023500	1.14142400
C	2.65626600	0.29075400	0.24301300
C	4.95226300	0.89900200	0.74641300
C	1.28364400	0.16586500	0.64388200
C	3.04444700	-0.08608400	-1.07106900
C	5.33994400	0.53283000	-0.56473100
H	5.69654900	1.27331400	1.44296300
C	0.33345000	-0.31242900	-0.24953500
C	2.06005100	-0.58451400	-1.96593900
C	4.40717000	0.05080200	-1.45044300
H	6.37835500	0.63368000	-0.86667300
C	0.75803500	-0.68424600	-1.55627200
H	2.34685900	-0.86735500	-2.97476400
H	4.69755800	-0.23437200	-2.45839100
H	0.00986500	-1.03688100	-2.26224800
H	3.34132200	1.05690700	2.14606300
O	1.00286100	0.57731800	1.89571700
H	0.22830300	0.07696000	2.28079400

Structure and coordinates of 2-TS-p6-2w.log

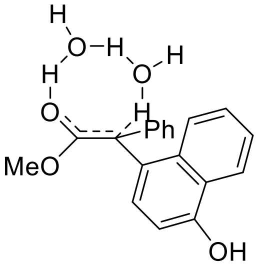

C	-3.37078100	2.55915700	0.32796800
C	-2.74501600	1.32778400	0.49883700
C	-1.67621900	0.92956300	-0.32159500
C	-1.25118600	1.82843800	-1.31096000
C	-1.86825400	3.06772500	-1.47918900
C	-2.93400300	3.44111100	-0.66258100
H	-4.19494600	2.83771800	0.97932700
H	-3.08542500	0.65909700	1.28489700

H	-0.42091600	1.55598100	-1.95703800
H	-1.51374200	3.74257600	-2.25363700
H	-3.41551300	4.40606700	-0.79132800
C	-0.97806500	-0.37777300	-0.11399300
C	-1.73782900	-1.57578200	-0.23164900
O	-1.30241800	-2.75591000	-0.10266900
O	-3.05754600	-1.41560300	-0.45253900
C	-3.87946300	-2.58025400	-0.38133000
H	-3.61477500	-3.29644300	-1.16280400
H	-3.79567900	-3.06120300	0.59688400
H	-4.89835800	-2.22295000	-0.53378800
O	0.38054100	-3.05418700	1.70669800
H	0.17034400	-3.84971600	2.21608100
H	-0.22864200	-3.02721700	0.86578100
O	-0.50597600	-0.99039800	2.52573500
H	-0.78756000	-0.63546700	1.56454800
H	-0.00061300	-2.03066600	2.25168400
H	0.14104600	-0.36856700	2.89333300
C	3.78482900	1.15465600	0.44681100
C	2.83079000	0.31319200	-0.18537400
C	3.39822100	2.07675900	1.38717900
C	3.23836500	-0.64284500	-1.16087900
C	1.44745200	0.41608800	0.15424400
C	2.03198400	2.19778200	1.72635600
H	4.13558700	2.71577100	1.86336900
C	2.30640300	-1.44172500	-1.77267100
C	0.47673800	-0.44364300	-0.47679400
C	1.09132800	1.39598200	1.12580100
H	1.71988700	2.93564900	2.45947900
C	0.94224400	-1.32646500	-1.43076500
H	0.04745800	1.53953500	1.38192800
H	0.23928600	-1.97636100	-1.94110400
H	4.83000200	1.05538800	0.17412500
H	2.61303400	-2.16296200	-2.52611500
O	4.57049500	-0.70335600	-1.43883100
H	4.73352800	-1.37911100	-2.10928000

Structure and coordinates of 2-TS-o6-1w.log

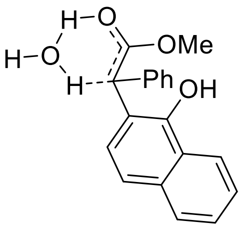

C	-2.03414000	3.01040200	-1.25199900
C	-1.37660100	1.79783700	-1.04643400
C	-1.84074300	0.87437500	-0.09911600
C	-2.98432500	1.21511100	0.64238800
C	-3.65171600	2.41709700	0.42890600
C	-3.17721600	3.32467900	-0.51995600
H	-1.65200900	3.70899800	-1.99097900
H	-0.48807900	1.56698800	-1.62778400
H	-3.35175800	0.52180600	1.39526400
H	-4.53753900	2.65267400	1.01224500
H	-3.68917700	4.26892900	-0.67977300
C	-1.13094400	-0.42007300	0.15920600
C	-1.86863400	-1.63223200	-0.04633300
O	-1.48604700	-2.76009000	0.40037400
O	-3.04446400	-1.54905000	-0.65797800
C	-3.88985000	-2.70798500	-0.63829600
H	-4.80445400	-2.40320500	-1.14545100
H	-3.42182000	-3.53758800	-1.17131900
H	-4.10942400	-3.00375000	0.39022100
H	-0.88240200	-2.44338300	1.55961800
O	-0.56302800	-1.74223000	2.35597600
H	-0.87703200	-0.90939900	1.69729300
H	-1.14984800	-1.81041000	3.12526200
C	1.22339600	0.37149000	0.49228500
C	2.62392300	0.34442500	0.22399900
C	0.33659200	-0.47675800	-0.15319300
C	3.53185400	1.21233000	0.88855400
C	3.12271900	-0.59041100	-0.72461500
C	0.86962900	-1.37958700	-1.11586100
C	4.87855400	1.14851900	0.62205500
H	3.14868200	1.92568300	1.61013900
C	4.52033700	-0.62827500	-0.97812900
C	2.20930900	-1.44982000	-1.39197100
H	0.18303000	-2.03401000	-1.64505100
C	5.37947000	0.21828600	-0.32078900
H	5.56454900	1.81498600	1.13634900
H	4.89783300	-1.34393500	-1.70391300
H	2.58375600	-2.15363800	-2.12996600
H	6.44582800	0.17685600	-0.52200600
O	0.82259200	1.27374900	1.42858500
H	-0.14223800	1.32691600	1.44998400

Structure and coordinates of 1-methoxynaphthalene.log

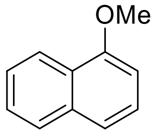

C	2.17785700	-1.74566000	0.00000200
C	0.81195100	-1.59012200	-0.00010300
C	0.23394000	-0.29409200	-0.00010600
C	1.08110800	0.84886100	-0.00000800
C	2.48855700	0.65194100	0.00008900
C	3.02545700	-0.61260500	0.00009800
H	2.61139100	-2.74110700	0.00004300
H	0.16041900	-2.45712500	-0.00015500
C	-1.18674900	-0.09737700	-0.00013000
C	0.50742800	2.15204200	0.00004000
H	3.13657200	1.52452400	0.00017900
H	4.10298500	-0.74739600	0.00016800
C	-0.85427500	2.29894500	-0.00002400
C	-1.71695000	1.17373300	-0.00009800
H	1.16292800	3.01831000	0.00011500
H	-1.29641100	3.29078900	0.00000200
H	-2.78855500	1.33354200	-0.00017000
O	-1.92189400	-1.23454000	-0.00021900
C	-3.33690000	-1.12005400	0.00029200
H	-3.71927300	-2.14087000	0.00058300
H	-3.69138100	-0.59886100	0.89679000
H	-3.69205900	-0.59914900	-0.89611400

Structure and coordinates of 3-TS-p1.log

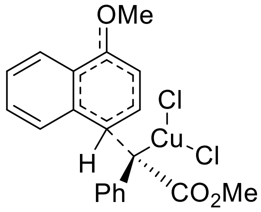

C	-2.65038900	-0.65254500	-2.60090300
C	-1.42321600	-0.41095500	-1.98887600
C	-1.22358600	0.73191200	-1.18656000
C	-2.30681700	1.62816900	-1.02362500
C	-3.52246000	1.39135100	-1.64056600
C	-3.70108100	0.24307300	-2.42231400
H	-2.78563700	-1.53912500	-3.21234100
H	-0.59720800	-1.10019700	-2.13486800
H	-2.18729600	2.51378000	-0.40755300
H	-4.34100200	2.09199900	-1.50854900

H	-4.65959800	0.05702000	-2.89770000
C	0.34346400	2.27647900	0.05425200
O	0.65170200	2.56402300	1.19570500
O	0.32888700	3.17884300	-0.94083100
C	0.72039300	4.51583000	-0.59683500
H	0.67279400	5.07685900	-1.52954000
H	0.02877300	4.93929900	0.13538800
H	1.73707200	4.52872000	-0.19763100
C	0.05284100	0.92873700	-0.52666400
Cu	1.67174600	0.10247100	-1.11699500
Cl	3.54299600	-0.82381200	-1.86853200
Cu	3.78330800	-0.73390700	0.33149900
Cl	3.99711600	-0.64863600	2.47192800
C	-4.08845300	-0.55722800	1.39715700
C	-2.71393200	-0.82696000	1.21623500
C	-4.48610700	0.59136900	2.04744600
C	-2.26566100	-2.02255500	0.54748700
C	-1.74273100	0.08551700	1.69565700
C	-3.52419400	1.50176100	2.52771900
H	-5.54254700	0.79558500	2.19085800
C	-0.90892800	-2.33321100	0.46515200
C	-0.34985200	-0.17137000	1.45058500
C	-2.17732700	1.25576800	2.35283600
H	-3.84608900	2.40334100	3.04028200
C	0.02430600	-1.42139500	0.94381200
H	-0.57345500	-3.26149600	0.02043600
H	0.38998000	0.45204300	1.93850000
H	-1.43389500	1.96015600	2.71246000
H	1.08050500	-1.66845500	0.88471900
H	-4.82306600	-1.26164900	1.02339500
O	-3.23266500	-2.79540800	0.06694900
C	-2.89006400	-3.98343500	-0.64857600
H	-2.25955400	-3.74609000	-1.51112600
H	-2.38285900	-4.69678400	0.00751400
H	-3.83601600	-4.40326400	-0.98768100

Structure and coordinates of 3-TS-o1.log

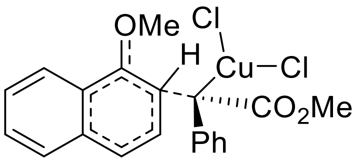

C	3.12067700	0.61335200	2.26262200
C	1.90014900	0.37858200	1.64897800

C	1.44041100	1.20020800	0.59698100
C	2.26863900	2.26040600	0.17102000
C	3.48724500	2.50377800	0.79315000
C	3.91695800	1.67810500	1.83305900
H	3.45575000	-0.03026400	3.06965900
H	1.27124700	-0.44538500	1.97718400
H	1.94707100	2.90251700	-0.64324500
H	4.10888400	3.32958900	0.46149700
H	4.87314000	1.86496600	2.31300700
C	-0.54711700	2.02174100	-0.74198000
O	-1.01495900	1.99727300	-1.86432300
O	-0.70889400	3.06495700	0.08863200
C	-1.55873300	4.12244500	-0.37911900
H	-1.57088100	4.85870600	0.42370800
H	-1.15495100	4.56563300	-1.29206500
H	-2.56747600	3.74522300	-0.56392000
C	0.14292600	0.92181700	0.00454600
Cu	-1.16019200	-0.03078800	1.03713800
Cl	-2.74803800	-0.93156100	2.29801900
Cu	-3.74895200	-0.64781100	0.34364300
Cl	-4.76192300	-0.40976300	-1.54011100
C	2.90907200	-2.56202800	0.16621200
C	2.44920900	-1.56947100	-0.73112000
C	4.23678900	-2.60611300	0.52853500
C	1.06769300	-1.45905800	-1.08292500
C	3.36300800	-0.62787800	-1.27739700
C	5.14550700	-1.66801300	-0.00332100
H	4.58556800	-3.35969100	1.22730400
C	0.61236500	-0.34784000	-1.81450400
C	2.90072900	0.36877200	-2.20296000
C	4.71930900	-0.70452900	-0.89370300
H	6.19078300	-1.70893800	0.28821300
C	1.57611000	0.48536700	-2.47401000
H	-0.41138300	-0.31652100	-2.16305500
H	3.62839200	1.02952800	-2.66404800
H	5.42247100	0.01501500	-1.30341000
H	1.20752100	1.25105300	-3.14710400
H	2.20244800	-3.27629200	0.57458200
O	0.27764200	-2.42450000	-0.63296900
C	-1.05441000	-2.55019600	-1.14388400
H	-1.66945700	-1.68455300	-0.87633000
H	-1.03100300	-2.67530900	-2.23014400
H	-1.46313100	-3.44191300	-0.67073300

Structure and coordinates of 3-Int-p2.log

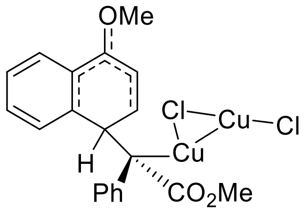

C	0.70749600	-0.89654600	2.94005700
C	0.19434900	-0.32044700	1.77730100
C	0.87297500	0.70526200	1.10430400
C	2.08898000	1.14249500	1.65498600
C	2.60031500	0.58299800	2.82231300
C	1.91524700	-0.44712600	3.46870400
H	0.15550500	-1.69147000	3.43393200
H	-0.75384100	-0.67677000	1.38170500
H	2.64674200	1.93198600	1.16158900
H	3.54103100	0.94890100	3.22460200
H	2.31737600	-0.88861200	4.37595100
C	0.66078600	2.64023900	-0.55278900
O	0.86387000	3.09526500	-1.67610200
O	0.62550000	3.44959800	0.52767500
C	0.79931000	4.84512600	0.28208900
H	0.71424900	5.32609300	1.25673100
H	1.78365100	5.04529900	-0.14953800
H	0.02429600	5.22333400	-0.38992600
C	0.37298300	1.22549600	-0.22822500
Cu	-1.61585500	1.35243500	-0.18125500
Cl	-3.83557900	1.50316800	-0.07934400
Cu	-3.52626500	-0.68385400	-0.05614700
Cl	-3.28455300	-2.82877400	-0.07990400
C	3.95266000	-1.77519100	-0.57117500
C	2.61578600	-1.41848000	-0.83528400
C	4.96016800	-0.84839700	-0.76128700
C	1.54648800	-2.38351600	-0.68367200
C	2.29177700	-0.11918600	-1.27242200
C	4.64449700	0.43675700	-1.22243200
H	5.99222700	-1.11758200	-0.56261900
C	0.22108500	-2.09869100	-1.10799300
C	0.85943600	0.29789800	-1.42354000
C	3.32743900	0.79757000	-1.47110300
H	5.43720900	1.16138700	-1.38235700
C	-0.07988100	-0.83397100	-1.49691000
H	-0.54610900	-2.86224100	-1.08683800
H	0.73976000	0.92198600	-2.31703700

H	3.08969400	1.80078000	-1.80999500
H	-1.09337600	-0.60753900	-1.81875400
H	4.18580100	-2.77788300	-0.23060500
O	1.88945100	-3.52817200	-0.17614600
C	0.91429900	-4.57109900	0.04215000
H	1.47205600	-5.38155000	0.50615300
H	0.13420000	-4.21318400	0.71670200
H	0.49553600	-4.89718200	-0.91164800

Structure and coordinates of 3-Int-o2.log

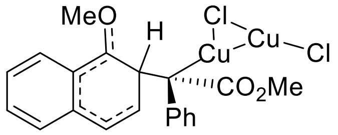

C	-2.91585800	0.94641800	-2.35613200
C	-1.72962900	0.72887200	-1.65693900
C	-1.45115800	1.39519200	-0.45556200
C	-2.41264600	2.30020200	0.01871600
C	-3.59566700	2.53346700	-0.67905600
C	-3.85576300	1.85261000	-1.86869400
H	-3.10045500	0.41175800	-3.28369300
H	-1.00278500	0.01868700	-2.04631900
H	-2.23532100	2.83044500	0.95019900
H	-4.31933100	3.24392600	-0.28912600
H	-4.78053900	2.02948000	-2.41027400
C	0.42701000	2.19992400	1.05927000
O	0.98679700	2.13769100	2.14955800
O	0.38203400	3.35473700	0.35958200
C	1.02968500	4.48102600	0.95121600
H	0.88913600	5.30068700	0.24601500
H	0.57369500	4.73201900	1.91255500
H	2.09657700	4.28937600	1.09364100
C	-0.20082300	1.06866900	0.33949200
Cu	1.32623200	0.61922900	-0.85566100
Cl	2.97096600	0.03041100	-2.22884800
Cu	3.53296800	-0.79351700	-0.24874700
Cl	4.13265200	-1.66604400	1.62899200
C	-2.51751700	-2.65332500	-0.60447800
C	-2.11403100	-1.77957000	0.43500100
C	-3.84917300	-2.96730800	-0.77938500
C	-0.76220700	-1.35764900	0.65080100
C	-3.10355200	-1.20459400	1.28856800
C	-4.81159600	-2.43968800	0.09300800
H	-4.14779200	-3.61879300	-1.59331600

C	-0.45853500	-0.10376900	1.39280300
C	-2.75039300	-0.23896800	2.31598300
C	-4.44267700	-1.57515700	1.11031600
H	-5.85763700	-2.69850000	-0.03915500
C	-1.51831900	0.28085500	2.36746900
H	0.49549400	-0.24308500	1.90833100
H	-3.53191500	0.08528300	2.99624200
H	-5.19523400	-1.15625100	1.77096800
H	-1.25813600	1.05612700	3.08013400
H	-1.80149300	-3.04213200	-1.31351800
O	0.31023800	-1.93695700	0.24248200
C	0.45213600	-3.19507500	-0.44815700
H	1.48761900	-3.47882500	-0.26953000
H	-0.22682000	-3.93995900	-0.03160200
H	0.28832800	-3.03671100	-1.51460100

Structure and coordinates of 3-TS-p3.log

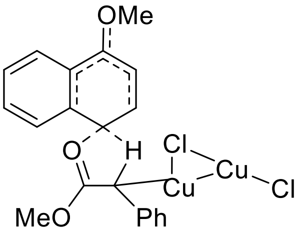

C	-1.64323500	2.84853600	-2.53759900
C	-1.21309700	1.82103200	-1.70454100
C	-0.50391900	2.08358800	-0.51690100
C	-0.24220500	3.42966500	-0.20498700
C	-0.67390400	4.45687000	-1.04323900
C	-1.37720300	4.17904000	-2.21379800
H	-2.18810900	2.60590300	-3.44579300
H	-1.42844000	0.79309600	-1.98310900
H	0.30423600	3.67966300	0.69571200
H	-0.45644300	5.48647100	-0.77229100
H	-1.71065200	4.98279700	-2.86324400
C	0.39129400	1.02559200	1.69777700
O	0.23704100	0.02559700	2.45465500
O	0.97648200	2.12385100	2.14107000
C	1.54795300	2.08141600	3.45997400
H	1.98939300	3.06577300	3.61024400
H	0.77477400	1.89304300	4.20689400
H	2.31862500	1.30945600	3.51079200
C	-0.07037500	0.92165900	0.32532600
Cu	1.73516100	0.63300700	-0.52177400
Cl	3.68069300	0.39228500	-1.56301700

Cu	3.44260900	-1.35703400	-0.21996900
Cl	3.05515600	-3.06867100	1.02797200
H	-0.48847900	-0.64681400	1.45588600
C	-0.96565200	-0.32825500	0.30500200
C	-2.40149100	-0.17085700	0.59253400
C	-0.59844400	-1.47004100	-0.45683100
C	-2.89860100	0.94986200	1.28980100
C	-3.31735500	-1.17888300	0.20863600
C	-1.47771600	-2.47377900	-0.81725000
H	0.44974800	-1.57662100	-0.72739100
C	-4.24239200	1.06483600	1.57848100
H	-2.21877900	1.73806900	1.59461500
C	-4.69269100	-1.04838600	0.51503000
C	-2.83112600	-2.33414700	-0.50286700
H	-1.10906900	-3.34484100	-1.34328900
C	-5.14937100	0.06092300	1.18938500
H	-4.60390300	1.94101300	2.10803200
H	-5.38071000	-1.83056400	0.21519600
H	-6.20457800	0.16306900	1.42110100
O	-3.75105600	-3.22584800	-0.82163000
C	-3.38392600	-4.41883600	-1.52645900
H	-2.69850900	-5.02090800	-0.92422900
H	-4.31708400	-4.95874800	-1.67796000
H	-2.93579600	-4.17231200	-2.49245000

Structure and coordinates of 3-TS-o3.log

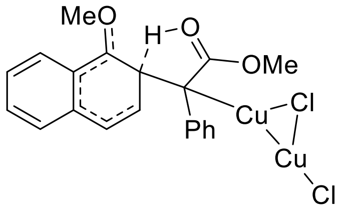

C	-0.77812500	4.04198600	-1.83124300
C	-0.75689200	2.83409000	-1.13985100
C	0.21734600	2.56151900	-0.16017800
C	1.18488700	3.55587200	0.07799300
C	1.16002200	4.76413900	-0.61606100
C	0.18037800	5.02118300	-1.57433800
H	-1.54926900	4.21365300	-2.57721600
H	-1.51308900	2.08939500	-1.37173200
H	1.96393000	3.38571900	0.81020600
H	1.91912100	5.51131900	-0.40112000
H	0.16534500	5.96511900	-2.11068000
C	0.83030400	0.97258500	1.81164900

O	0.33628800	0.09097200	2.57020400
O	1.91867800	1.63404600	2.16942000
C	2.56926200	1.22363400	3.38389700
H	3.41114700	1.90490500	3.49975200
H	1.88864600	1.31314000	4.23250700
H	2.92443900	0.19475200	3.29173200
C	0.17807400	1.23878300	0.54234300
Cu	1.48068400	0.20615900	-0.59892300
Cl	3.05916100	-0.75740200	-1.84352000
Cu	2.44485500	-2.27828300	-0.35794700
Cl	1.79398100	-3.76361700	1.05951900
C	-3.51381700	-2.13794300	-0.63275500
C	-3.04117100	-0.90686600	-0.10489700
C	-4.85164900	-2.46086400	-0.59167000
C	-1.66391200	-0.47630100	-0.15469900
C	-3.97900900	-0.03767900	0.53005300
C	-5.78005100	-1.56640700	-0.02934500
H	-5.18595300	-3.41352600	-0.98883600
C	-1.20678900	0.59010700	0.69340900
C	-3.54872400	1.15761200	1.20148800
C	-5.34685700	-0.38421400	0.52984200
H	-6.83584800	-1.81897700	-0.01550200
C	-2.23533400	1.44296500	1.29230200
H	-0.79520000	-0.03941300	1.72134000
H	-4.29735900	1.79442900	1.66214300
H	-6.05307600	0.29441200	0.99867700
H	-1.88728100	2.30854500	1.84848200
H	-2.82879700	-2.86736400	-1.03614200
O	-0.72612700	-0.99942000	-0.90626600
C	-0.84710600	-1.95956600	-1.96946500
H	-0.85289800	-2.97095000	-1.56164300
H	-1.72823800	-1.75520200	-2.57861900
H	0.05460300	-1.81363100	-2.56381700

Structure and coordinates of 3-Int-p4.log

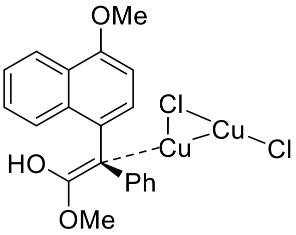

C	0.89876400	4.54620400	-0.65429600
C	1.00372600	3.20822300	-0.28424900
C	-0.11825900	2.36073100	-0.30054200

C	-1.33920400	2.90307300	-0.73029500
C	-1.44306500	4.24318300	-1.09880400
C	-0.32640300	5.07509400	-1.05994900
H	1.78364800	5.17584600	-0.63021200
H	1.97044600	2.82105600	0.01830700
H	-2.22388900	2.27675900	-0.77391200
H	-2.40493000	4.63436800	-1.41769600
H	-0.40800600	6.11953900	-1.34566400
C	0.70991600	0.49569600	1.20006600
O	0.47925100	-0.68882500	1.75308300
O	1.53862500	1.33850000	1.80723900
C	2.29115900	0.91431800	2.95443000
H	2.75970500	1.82122100	3.33289700
H	1.63217000	0.49055700	3.71537300
H	3.06074600	0.19520900	2.66385600
C	-0.04884800	0.90884700	0.09403400
Cu	1.54766200	0.17270000	-0.99392900
Cl	3.25569700	-0.69919000	-2.14323300
Cu	3.12240500	-1.73559100	-0.18811300
Cl	2.88936400	-2.56098300	1.81315900
C	-4.56518000	-0.94457000	1.23629600
C	-3.47741300	-0.84531900	0.33016700
C	-4.55004200	-0.26078400	2.42808900
C	-3.49685100	-1.53980500	-0.92438400
C	-2.34990800	-0.04305500	0.66455300
C	-3.44696500	0.55870700	2.75667700
H	-5.38518100	-0.34498300	3.11685900
C	-2.44324500	-1.41575600	-1.79869600
C	-1.24096200	0.04704600	-0.24619700
C	-2.37993600	0.66374800	1.89741700
H	-3.44284700	1.10842100	3.69317300
C	-1.32969100	-0.61814800	-1.44464300
H	-1.54886900	1.30909400	2.16386000
H	-0.51851300	-0.54111800	-2.16673800
H	-5.41147900	-1.56907500	0.97201400
H	1.29700700	-1.20200300	1.95547700
H	-2.44054500	-1.92152000	-2.75662300
O	-4.60168300	-2.28335900	-1.15474900
C	-4.65861400	-3.05393700	-2.34670000
H	-3.83288200	-3.77322000	-2.38925900
H	-5.60630800	-3.59155400	-2.30772700
H	-4.64169100	-2.41334600	-3.23593700

Structure and coordinates of 3-Int-o4.log

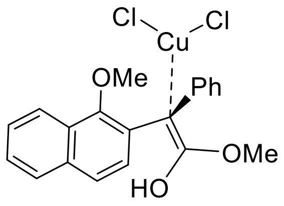

C	1.09823800	4.49751300	-0.52109400
C	1.15196400	3.22629300	0.04487800
C	0.00098500	2.41706900	0.12501200
C	-1.19230900	2.94032000	-0.40819000
C	-1.24003500	4.21131100	-0.97393100
C	-0.09537500	5.00351800	-1.03217700
H	2.00667300	5.09162400	-0.56629900
H	2.10031900	2.86241700	0.41585500
H	-2.10212800	2.35099200	-0.38724400
H	-2.18106600	4.58011000	-1.37216400
H	-0.13173000	5.99383500	-1.47553100
C	0.97899400	0.52429100	1.54502500
O	0.76674900	-0.61870100	2.19900800
O	2.05749700	1.23720400	1.85345700
C	3.04705400	0.71120000	2.75134500
H	3.71438700	1.54733200	2.95395100
H	2.58606400	0.36898600	3.68001300
H	3.60365300	-0.09912300	2.27443200
C	-0.00505300	1.02028800	0.68909300
Cu	1.03326700	0.17581800	-0.86473500
Cl	2.50782700	-0.53397800	-2.39592600
Cu	2.80287700	-1.70026400	-0.54039200
Cl	2.96899300	-2.72376800	1.36599700
C	-3.81478100	-1.61624100	-1.25034900
C	-3.22917700	-0.89349000	-0.17513700
C	-5.13746000	-1.98572600	-1.20434600
C	-1.86116900	-0.47933700	-0.18286300
C	-4.04342700	-0.52201400	0.93073900
C	-5.93742000	-1.65614800	-0.08359000
H	-5.57656100	-2.52592200	-2.03769500
C	-1.35660900	0.35957400	0.78497700
C	-3.48706000	0.28038600	1.96435500
C	-5.40271000	-0.93531800	0.95566000
H	-6.97908800	-1.96155800	-0.05708300
C	-2.19687300	0.72580600	1.87392900
H	-4.10934200	0.55887200	2.81030900
H	-6.01533100	-0.65776800	1.80943200
H	-1.78990400	1.38070200	2.63965600
H	-3.21850500	-1.85139100	-2.12586900

O	-1.04678100	-0.89450500	-1.21752300
H	1.52243800	-1.24349300	2.11823000
C	-0.66476400	-2.28161500	-1.12663500
H	-1.53837300	-2.93765700	-1.15516400
H	-0.02964800	-2.48315600	-1.98964400
H	-0.11376700	-2.46227200	-0.19743300

Structure and coordinates of 3/4-Int-p5.log

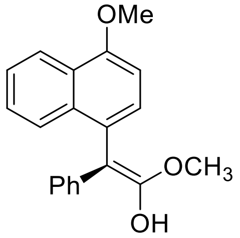

C	-2.52399500	1.68705200	2.35636400
C	-1.75535900	0.70388100	1.73830500
C	-2.02504200	0.29529000	0.42435500
C	-3.07563400	0.92487500	-0.26005800
C	-3.84733700	1.90647300	0.35731200
C	-3.57754300	2.29115800	1.67056000
H	-2.29909200	1.98092500	3.37764600
H	-0.93289700	0.24521200	2.27991200
H	-3.28278600	0.64715300	-1.29016800
H	-4.65592700	2.37896600	-0.19314800
H	-4.17546000	3.06003500	2.15073700
C	-1.16773400	-0.72797900	-0.22914000
C	-1.65395200	-1.70352700	-1.02387700
O	-2.94133100	-2.06757100	-1.21073000
C	-3.80949800	-2.19127500	-0.07776600
H	-4.51311900	-2.98712900	-0.32601500
H	-3.23942000	-2.46180900	0.81540600
H	-4.35389100	-1.26139700	0.09880600
C	0.93967200	-1.52934900	0.82999900
C	0.30596700	-0.63938700	-0.00245200
C	1.08493000	0.40472900	-0.60472900
C	2.47586900	0.50925800	-0.31957500
C	3.07955300	-0.44341600	0.56528500
C	2.32291900	-1.44825200	1.12062300
H	-0.54354700	1.27010300	-1.73712600
H	0.36173800	-2.32425300	1.29349900
C	0.51270500	1.34794900	-1.50090700
C	3.23833900	1.54495100	-0.91903700
C	2.64957200	2.44422200	-1.77553100
C	1.27164300	2.34103300	-2.07149500

H	4.29681000	1.61574200	-0.69400800
H	3.24276600	3.23221100	-2.22964700
H	0.81257600	3.04861800	-2.75563100
O	4.40375000	-0.26429100	0.78737000
O	-0.83352800	-2.44606800	-1.79926000
H	-1.32791700	-3.21804200	-2.10873600
H	2.76051900	-2.17912700	1.79023500
C	5.06473400	-1.16372100	1.66397600
H	6.10340800	-0.83436400	1.70161800
H	4.63377600	-1.12266000	2.67081300
H	5.02246900	-2.19053200	1.28350100

Structure and coordinates of 3/4-Int-o5.log

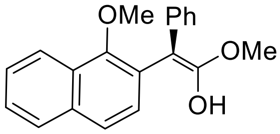

C	-2.54560300	-2.64789700	-1.24601100
C	-1.71462200	-1.58610900	-0.89839500
C	-2.09951900	-0.65933000	0.08167300
C	-3.33658600	-0.84493700	0.71753000
C	-4.17064200	-1.90433800	0.36696900
C	-3.78023400	-2.81110900	-0.61779000
H	-2.22615700	-3.35026900	-2.01047200
H	-0.75729700	-1.46887600	-1.39708800
H	-3.64446700	-0.15354000	1.49713900
H	-5.12373200	-2.02707000	0.87359300
H	-4.42774400	-3.64017400	-0.88739100
C	-1.18383600	0.44027900	0.47538100
C	-1.59931300	1.68786900	0.76848100
O	-2.81753700	2.23045200	0.56028800
C	-3.45765700	2.02734000	-0.70670900
H	-3.97976800	2.95789800	-0.93502800
H	-2.71543300	1.81403400	-1.48044900
H	-4.17782300	1.20843600	-0.64942100
C	0.74025800	-0.42882300	1.85872200
C	2.05532000	-0.77076000	2.03228500
C	2.98628800	-0.60982400	0.97039900
C	2.53130400	-0.09333000	-0.27500100
C	1.15555300	0.26572200	-0.40975500
C	0.26447900	0.10746700	0.63060900
H	4.70145600	-1.35508000	2.06082600
H	0.02910500	-0.56006000	2.66950500
H	2.40129500	-1.17233400	2.98081400

C	4.35653100	-0.96144400	1.10833100
C	3.45351700	0.04267500	-1.34758700
C	4.77155600	-0.30709800	-1.18218800
C	5.23020400	-0.81112600	0.05961600
H	3.09898100	0.41974900	-2.30133700
H	5.46942300	-0.20091200	-2.00738500
H	6.27526800	-1.08137200	0.17744900
O	-0.77131500	2.57386400	1.36719400
H	-1.16300600	3.45533600	1.28989100
O	0.72341200	0.71297400	-1.63420600
C	0.79744900	2.12444800	-1.81739700
H	0.12749400	2.64721800	-1.12658200
H	1.82075400	2.49019800	-1.66974300
H	0.48849000	2.32045000	-2.84549600

Structure and coordinates of 3-Int-p5*.log

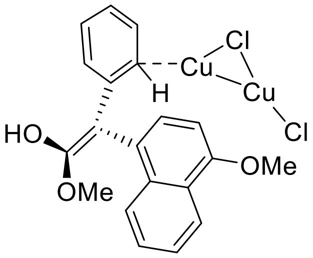

C	-3.42843300	1.37955100	1.59313900
C	-2.43329700	1.83513500	0.72318500
C	-1.08384800	1.52592000	0.93260600
C	-0.76185600	0.72516900	2.06358700
C	-1.76760000	0.26780600	2.93930700
C	-3.11370000	0.59396800	2.69411900
H	-4.46407100	1.63328100	1.38881000
H	-2.72698300	2.41833800	-0.13994000
H	0.28107300	0.55875700	2.32138300
H	-1.48979700	-0.25676100	3.84990100
H	-3.88813900	0.24420000	3.36848900
C	0.01629300	1.95914700	0.04409500
C	-0.00807700	3.09834600	-0.67788500
O	0.98616900	3.38118900	-1.54844200
O	-0.97446200	4.02792800	-0.56464400
C	-1.69808000	4.33243300	-1.77184600
H	-2.58759200	4.87731900	-1.45574200
H	-1.10695100	4.96319800	-2.44230300
H	-1.98697100	3.40964800	-2.28497400
C	2.26538900	-2.25756800	-1.32183200
C	2.30718600	-0.99324800	-0.67202900

C	3.35834400	-3.08881300	-1.30758700
C	1.18639800	-0.12230500	-0.66394200
C	3.50420100	-0.60295600	-0.00781300
C	4.54673600	-2.69736100	-0.64124000
H	3.31458700	-4.05335000	-1.80445200
C	1.23287800	1.09254800	-0.02003100
C	3.53113300	0.66981500	0.63564200
C	4.62056500	-1.48123000	-0.00611100
H	5.40177900	-3.36692600	-0.63392400
C	2.42966200	1.48860900	0.63321600
H	5.52667500	-1.17937400	0.50933800
H	2.47643000	2.44610500	1.14437400
H	1.35107300	-2.55386400	-1.82937400
Cu	-1.32133200	-1.23918200	1.42534800
Cl	-1.54271300	-3.19182000	0.39040700
Cu	-2.21009700	-1.70904800	-1.10433000
Cl	-2.88132800	-0.28884600	-2.57675200
H	1.02465600	4.33524600	-1.70607600
O	4.67176400	1.04752000	1.29860500
H	0.27839600	-0.43255700	-1.17609300
C	5.53671300	1.88650100	0.53383000
H	5.85926400	1.38272300	-0.38550000
H	5.03928900	2.82879200	0.27539600
H	6.40601100	2.09334200	1.16042100

Structure and coordinates of 3-Int-o5*.log

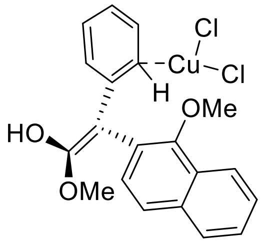

C	-2.67765700	0.92438200	2.68816500
C	-1.71915900	1.55724800	1.89282400
C	-0.51333500	0.92593900	1.54057900
C	-0.31351400	-0.39295300	2.04447000
C	-1.27193300	-1.01714400	2.87305500
C	-2.47393100	-0.35626700	3.18544700
H	-3.59437600	1.45615400	2.92485600
H	-1.91679800	2.56182600	1.54789600
H	0.64043800	-0.88835700	1.89055700
H	-1.03367300	-1.96827900	3.34364000
H	-3.20969300	-0.83424500	3.82333300
C	0.55186000	1.54467500	0.72921300

C	0.42829100	2.71082400	0.04323800
O	1.45024900	3.16282700	-0.70642600
O	-0.66542000	3.48231300	0.09242700
C	-1.11646100	4.09644300	-1.12880800
H	-2.16716400	4.33208100	-0.96462800
H	-0.57082000	5.02466400	-1.32801300
H	-1.02302400	3.40145600	-1.96608500
C	4.14370900	-0.91304000	-1.73695200
C	3.64258400	-0.33168700	-0.54146900
C	5.39092900	-1.48992500	-1.76088800
C	2.35154900	0.27793500	-0.48654100
C	4.44903800	-0.34523800	0.63069100
C	6.19454700	-1.51005600	-0.59444300
H	5.76819700	-1.92984600	-2.67929400
C	1.88337600	0.86958700	0.66879600
C	3.94470900	0.24794400	1.82047100
C	5.73346700	-0.95095800	0.57279200
H	7.17824100	-1.96891000	-0.62788900
C	2.70830500	0.83755200	1.82924100
H	4.55781900	0.23857000	2.71765800
H	6.34726100	-0.95961300	1.46997500
H	2.33184400	1.29935500	2.73825900
H	3.52860900	-0.88800600	-2.63046100
Cu	-1.63164600	-1.66658600	0.87495100
Cl	-2.55113600	-2.90648000	-0.73512700
Cu	-2.89558300	-0.82300300	-1.37752200
Cl	-3.27415000	1.19521900	-2.01681400
O	1.61597100	0.29495800	-1.64201700
H	1.36147900	4.11184900	-0.87031700
C	0.63096900	-0.72848200	-1.72277700
H	1.08814800	-1.72453500	-1.66154300
H	-0.10860600	-0.62377900	-0.91889100
H	0.13116200	-0.61233400	-2.68601900

Structure and coordinates of 3/4-TS-p6-2w.log

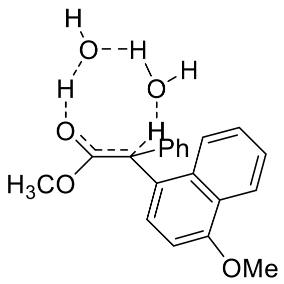

C	-3.89189400	2.22436100	-0.07951700
C	-3.15174900	1.08860100	0.23556400

C	-1.96911800	0.77105100	-0.45329700
C	-1.55068000	1.65358600	-1.46019100
C	-2.28313500	2.79885600	-1.77213700
C	-3.46073100	3.09037400	-1.08642800
H	-4.80464800	2.44022800	0.46953400
H	-3.49189200	0.43228700	1.03196800
H	-0.63327900	1.44468800	-2.00392300
H	-1.93088900	3.46372700	-2.55619300
H	-4.03194900	3.98227100	-1.32727900
C	-1.15630300	-0.43406600	-0.09615800
C	-1.76727100	-1.71499800	-0.20373800
O	-1.22271600	-2.83040600	0.03815100
O	-3.06994600	-1.71684400	-0.54956100
C	-3.77245000	-2.95461000	-0.45163900
H	-3.38866500	-3.68417500	-1.16925400
H	-3.70489900	-3.36767100	0.55841300
H	-4.81069300	-2.71709300	-0.68604600
O	0.30141000	-2.84072500	2.00601300
H	0.12883000	-3.62773600	2.54196200
H	-0.22171200	-2.92709000	1.11284400
O	-0.88412300	-0.85342300	2.60824600
H	-1.10756400	-0.58235700	1.60539000
H	-0.24249100	-1.83953800	2.44827600
H	-0.35226600	-0.14159200	2.99662800
C	3.33509200	1.64595800	0.81075100
C	2.54488600	0.67354500	0.14350900
C	2.76226300	2.56668000	1.65360000
C	3.15212700	-0.28119900	-0.73382700
C	1.13297100	0.64088000	0.34681100
C	1.36510800	2.55308400	1.85596200
H	3.37825000	3.30556500	2.15707000
C	2.37108100	-1.20600400	-1.38240700
C	0.32523800	-0.35291100	-0.31930500
C	0.57955800	1.62266600	1.21806500
H	0.90570800	3.28744700	2.51119200
C	0.97362200	-1.22159100	-1.17027000
H	-0.49396400	1.66081600	1.36705300
H	0.39591400	-1.97077200	-1.70167900
H	4.40630300	1.65130500	0.64226200
H	2.80233200	-1.93034100	-2.06357800
O	4.49945500	-0.18279700	-0.85834100
C	5.15651300	-1.08902700	-1.72962200
H	6.21734000	-0.84340700	-1.67330400
H	5.00726300	-2.12664900	-1.40946200

H	4.80958100	-0.96777000	-2.76228900

Structure and coordinates of 3/4-TS-o6-2w.log

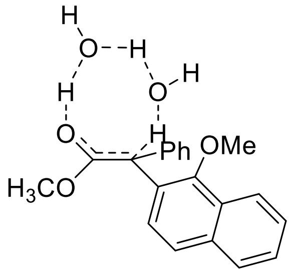

C	-3.46922900	2.67877100	0.33727900
C	-2.90085100	1.41309700	0.45105600
C	-1.78223500	1.03933900	-0.30985300
C	-1.24599300	1.99873100	-1.18074700
C	-1.80537000	3.27054600	-1.29343400
C	-2.92377300	3.61925100	-0.53760200
H	-4.33687700	2.93457700	0.93982000
H	-3.33548700	0.69547300	1.14208500
H	-0.37301600	1.74787700	-1.77745200
H	-1.36688900	3.98988400	-1.97994300
H	-3.36315700	4.60856000	-0.62606100
C	-1.17846800	-0.32420500	-0.18249000
C	-1.98514300	-1.45475000	-0.48630500
O	-1.63323800	-2.66691000	-0.39972100
O	-3.25694700	-1.19406800	-0.84706700
C	-4.15266100	-2.30047300	-0.94215000
H	-5.11397400	-1.86858400	-1.22164000
H	-3.82674800	-3.00978700	-1.70623100
H	-4.24292800	-2.81686900	0.01780800
O	-0.19988800	-3.16399500	1.57422700
H	-0.43727700	-4.00756200	1.98370300
H	-0.70350900	-3.06438300	0.67200000
O	-1.08658700	-1.12198200	2.44220800
H	-1.20564800	-0.68185700	1.48741800
H	-0.64348000	-2.18588800	2.15619200
H	-1.95963300	-1.18547100	2.85746300
C	3.51939200	1.23333300	0.59220600
C	2.59896700	0.32731300	-0.00431100
C	4.85961900	1.17692600	0.29216100
C	1.19959600	0.34321000	0.29334500
C	3.09166400	-0.60999700	-0.95558600
C	5.35325700	0.21478100	-0.62072600
H	5.54541900	1.88269500	0.75161500
C	0.29432400	-0.44893000	-0.40043200

C	2.16512000	-1.44887700	-1.62889500
C	4.48344000	-0.65309200	-1.23455400
H	6.41532300	0.17564200	-0.84349500
C	0.82471000	-1.34484200	-1.37778300
H	2.53125500	-2.15226400	-2.37211000
H	4.84618100	-1.38157400	-1.95521500
H	0.13473300	-1.96942400	-1.93408900
H	3.15710600	1.99175900	1.27592500
O	0.72002100	1.23032200	1.22251600
C	1.11030200	1.04195100	2.58374100
H	1.80837100	1.82601600	2.89341500
H	0.20881000	1.10856100	3.19658400
H	1.57451600	0.06318300	2.73538800

Structure and coordinates of 4-TS-p1.log

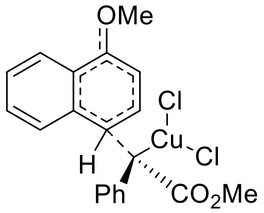

C	-1.62378700	0.86215800	2.54261400
C	-0.53948100	0.77838400	1.67674100
C	-0.13405900	-0.46836300	1.13986900
C	-0.87175500	-1.62680700	1.50561800
C	-1.95153600	-1.53479500	2.35997000
C	-2.33501700	-0.28761500	2.87432400
H	-1.91817600	1.82381400	2.94956200
H	0.01796400	1.67512400	1.42421200
H	-0.58974500	-2.59549700	1.10596200
H	-2.50915100	-2.42681400	2.62633000
H	-3.18700500	-0.22177200	3.54432200
C	0.95387900	-0.53704500	0.21095400
C	1.50914100	-1.89543300	-0.10952200
O	1.49412000	-2.47603700	-1.17434300
O	2.08983400	-2.37571700	0.99125300
C	2.79586200	-3.61884200	0.84947900
H	3.23924000	-3.80969800	1.82562100
H	2.10182700	-4.41862600	0.58141900
H	3.57444000	-3.52867200	0.08913000
Cu	2.21617300	0.96737500	0.04399500
Cl	2.31187800	3.21165700	0.16109900
Cl	4.12827600	-0.03587400	-0.54984300
C	-1.12772700	1.99166700	-1.20845200

C	-2.30771500	1.39901100	-0.76373000
C	-2.54613000	-0.01023500	-0.95440700
C	-1.51681500	-0.80173600	-1.52215900
C	-0.25718400	-0.19542100	-1.84347400
C	-0.13580700	1.19506000	-1.77317100
H	-4.55411600	0.00672600	-0.15259300
H	-0.96446500	3.05689000	-1.11001000
C	-3.77242000	-0.60581100	-0.58758400
C	-1.74343100	-2.18200800	-1.70552500
H	0.48721200	-0.77741900	-2.37324300
H	0.77144800	1.66666000	-2.14066700
C	-2.95091700	-2.74769100	-1.34781300
C	-3.97100800	-1.95616900	-0.78627500
H	-0.94905100	-2.78985200	-2.12672600
H	-3.11631700	-3.80982500	-1.50066100
H	-4.91590200	-2.41198800	-0.50765100
O	-3.29886800	2.06551500	-0.18193100
C	-3.15672200	3.46817300	0.05243300
H	-2.29024800	3.66962100	0.68947100
H	-3.06530800	4.00818100	-0.89451600
H	-4.06918300	3.77199000	0.56366400

Structure and coordinates of 4-TS-o1.log

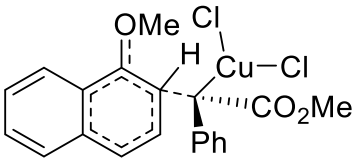

C	-1.82403300	0.65015700	-2.56231000
C	-0.80371400	0.23678400	-1.72443400
C	-0.25771900	1.10422800	-0.74634600
C	-0.81725700	2.40034800	-0.62623700
C	-1.84064500	2.81302100	-1.46839800
C	-2.34761900	1.93979000	-2.43129600
H	-2.22021000	-0.02983400	-3.30910000
H	-0.40438800	-0.76918700	-1.82363200
H	-0.43453400	3.08612600	0.12054500
H	-2.25448300	3.81099800	-1.36579500
H	-3.15148500	2.26426000	-3.08534500
C	0.80832300	0.63127300	0.09966200
C	1.67760500	1.62556800	0.82608200
O	2.08734800	1.52998300	1.96571900
O	2.03538200	2.60687700	-0.00235800
C	3.04751200	3.50380900	0.47767500

H	3.24549900	4.18546500	-0.34828900
H	2.68410300	4.05514600	1.34801800
H	3.95178700	2.94944000	0.73800200
Cu	1.80876000	-0.97001000	-0.50787100
Cl	1.77741500	-3.07216500	-1.27164100
Cl	3.84704400	-0.07480500	-0.79050200
C	-0.30524700	-0.02665100	2.06584300
C	-0.95877600	-1.11757400	1.46702900
C	-2.23189900	-0.93695500	0.84013000
C	-2.88801300	0.31272100	1.00140200
C	-2.28721800	1.34407200	1.80359800
C	-1.04698200	1.16804700	2.32743100
H	-2.30838100	-2.89868000	-0.06884600
H	0.62269600	-0.18032100	2.59831800
C	-2.82246500	-1.95129500	0.05221200
C	-4.12812500	0.50960500	0.36054200
H	-2.83951600	2.26399800	1.96866500
H	-0.56811100	1.95050700	2.90626600
C	-4.68465900	-0.48767600	-0.41560500
C	-4.03006900	-1.72539800	-0.57240700
H	-4.63501900	1.46285000	0.47894200
H	-5.63638100	-0.31862400	-0.91043500
H	-4.47634400	-2.49917800	-1.18866700
O	-0.45136100	-2.33905800	1.41469900
C	0.72860700	-2.64125900	2.16760000
H	1.57605000	-2.02576600	1.84818500
H	0.53937300	-2.48788000	3.23394000
H	0.94289800	-3.68866000	1.96639300

Structure and coordinates of 4-Int-p2.log

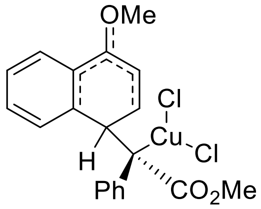

C	0.94326800	-1.45716100	2.79918900
C	0.22078700	-1.15862800	1.64432500
C	0.00860000	0.16790800	1.24073300
C	0.54460700	1.18979000	2.04219300
C	1.26526100	0.89535100	3.19514200
C	1.47232100	-0.43106900	3.57773600
H	1.08575300	-2.49419900	3.08864500
H	-0.19237100	-1.97321100	1.05634500

H	0.40741200	2.22870500	1.75732800
H	1.67138300	1.70542400	3.79398800
H	2.03528300	-0.65934100	4.47794600
C	-1.35878200	1.80330300	-0.15936900
O	-1.34291100	2.56276800	-1.11329300
O	-2.09518600	2.04941400	0.93726700
C	-2.89855000	3.23360000	0.91555200
H	-3.40489700	3.25933000	1.87985200
H	-2.27502200	4.12250000	0.79332200
H	-3.63167600	3.18351800	0.10660900
C	-0.66533000	0.48219600	-0.07559700
Cu	-2.30977000	-0.71861200	-0.18600200
C	4.02062400	0.77814200	-0.48442500
C	2.79124900	0.16421400	-0.79633900
C	4.11659700	2.15591600	-0.47076100
C	2.69849400	-1.27427000	-0.89541600
C	1.64505400	0.93865800	-1.07214500
C	2.99020400	2.92920800	-0.77947100
H	5.06189000	2.63512800	-0.23946500
C	1.52469200	-1.91061200	-1.39242000
C	0.31043500	0.27971200	-1.29934300
C	1.77222900	2.33041000	-1.07127900
H	3.06848800	4.01211100	-0.79294500
C	0.41280800	-1.16622300	-1.59261900
H	1.51534200	-2.97724500	-1.57591900
H	-0.20390000	0.75718300	-2.14351900
H	0.90604400	2.93963400	-1.30126600
H	-0.49100200	-1.65523900	-1.94664800
H	4.88965900	0.16563600	-0.27278500
O	3.75770500	-1.93341300	-0.54472500
Cl	-3.00814900	-0.32298100	-2.29080900
Cl	-3.53700300	-2.27334400	0.87401700
C	3.80689100	-3.37683500	-0.59795100
H	4.79752000	-3.63272300	-0.22893200
H	3.03863500	-3.79524300	0.05470600
H	3.68676600	-3.71651200	-1.62814300

Structure and coordinates of 4-Int-o2.log

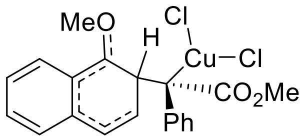

C	1.15401700	1.43214800	2.69476700
C	0.34338400	0.72045600	1.81269900

C	-0.01177300	1.25254000	0.56707800
C	0.46974300	2.52596800	0.23211900
C	1.27989500	3.24107600	1.11103800
C	1.62830400	2.69523900	2.34611300
H	1.40875600	0.99763900	3.65697700
H	-0.02863000	-0.25736400	2.10383900
H	0.21821700	2.96358000	-0.72979600
H	1.64178400	4.22510700	0.82728200
H	2.25983000	3.25281100	3.03144400
C	-1.75644500	1.16453200	-1.29504300
O	-1.99814000	0.92612200	-2.46805600
O	-2.41391300	2.10246400	-0.59995000
C	-3.46544300	2.78521100	-1.28984800
H	-3.87204300	3.49492000	-0.57002200
H	-3.07472100	3.31371200	-2.16283300
H	-4.23841600	2.07879600	-1.60223500
C	-0.76546700	0.42781800	-0.45498500
Cu	-2.12121000	-0.88264700	0.38303100
C	3.38317600	-1.37926700	0.67842800
C	2.54667800	-0.87984400	-0.35412100
C	4.71383700	-1.02459100	0.73958600
C	1.15418000	-1.18391800	-0.47613000
C	3.09564100	0.03614500	-1.30306200
C	5.25768900	-0.17129600	-0.23112700
H	5.33568900	-1.40381500	1.54295200
C	0.23592200	-0.38646900	-1.35748300
C	2.27080500	0.66036600	-2.32044300
C	4.45799500	0.35259300	-1.23229800
H	6.30982100	0.09249300	-0.18746400
C	0.94510100	0.49029800	-2.33543800
H	-0.36812100	-1.11250600	-1.91797000
H	2.76287900	1.30463800	-3.04267600
H	4.87493100	1.03443800	-1.96666700
H	0.32004300	0.99289400	-3.06410600
H	2.99247500	-2.00922100	1.46242700
Cl	-2.56161600	-2.47064200	-1.11732700
Cl	-3.00312300	-0.33185400	2.33883700
O	0.50677400	-2.11753200	0.12512400
C	0.99346800	-3.19764600	0.94744900
H	0.18264100	-3.92348500	0.93420400
H	1.89728700	-3.62699600	0.51470800
H	1.15122800	-2.83502800	1.96363200

Structure and coordinates of 4-TS-p3.log

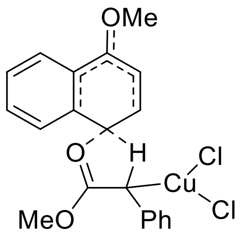

C	0.71950300	2.06616800	-2.76097500
C	0.33083500	1.21761300	-1.73526300
C	1.00783200	1.21636200	-0.49052000
C	2.09269300	2.11235500	-0.32949500
C	2.46861800	2.96046000	-1.36261200
C	1.78977700	2.94378900	-2.58213500
H	0.18725700	2.03993600	-3.70662500
H	-0.49814900	0.53577000	-1.89692200
H	2.63774500	2.13816600	0.60506300
H	3.30236200	3.63986600	-1.21435600
H	2.09397600	3.60713300	-3.38565600
C	0.95510300	0.30186000	1.95944700
O	0.17732600	-0.26169100	2.76799900
O	2.06068200	0.87955800	2.34687600
C	2.41827300	0.76471900	3.74098700
H	3.36298700	1.29699100	3.83444600
H	1.65233100	1.22835900	4.36473200
H	2.54352600	-0.28738100	4.00141000
C	0.57911900	0.25611500	0.54388800
Cu	1.95627200	-0.96365000	-0.38191900
H	-0.71268900	-0.57376900	1.78552200
C	-0.84776000	-0.29075300	0.51234300
C	-1.96750400	0.66134800	0.50349500
C	-1.10273600	-1.58709600	-0.00695900
C	-1.81595900	1.99369200	0.94038600
C	-3.25100200	0.23435500	0.08713700
C	-2.35685400	-2.01963100	-0.39511200
H	-0.27490400	-2.29055900	-0.05219400
C	-2.89059700	2.85820400	0.95598800
H	-0.84333600	2.35037100	1.26218500
C	-4.34362800	1.13287000	0.11200600
C	-3.42678700	-1.12270300	-0.36515600
H	-2.48947000	-3.03970100	-0.73085600
C	-4.16475200	2.42877900	0.53920800
H	-2.75001600	3.88127100	1.29101900
H	-5.32067600	0.78854800	-0.20729600
H	-5.00171200	3.11924700	0.55588300
O	-4.65345900	-1.43709900	-0.73653100

Cl	3.15353800	-1.34686800	-2.24380400
Cl	2.27976300	-2.58777400	1.15306700
C	-4.94928000	-2.76261300	-1.19663400
H	-4.74472600	-3.49397400	-0.41039400
H	-6.01338300	-2.75381300	-1.42687100
H	-4.37486800	-2.99318500	-2.09782000

Structure and coordinates of 4-TS-o3.log

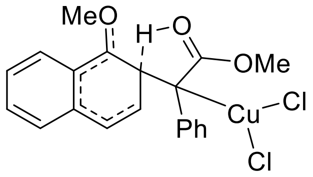

C	1.23889700	3.37125300	-1.45678700
C	0.63696500	2.32583300	-0.76933300
C	1.38188900	1.50313800	0.10541100
C	2.76414300	1.76852500	0.24228700
C	3.35652900	2.81778700	-0.45059900
C	2.60207900	3.62514000	-1.30187900
H	0.64038600	3.98654000	-2.12162500
H	-0.42151200	2.13838700	-0.92014100
H	3.37344900	1.14702300	0.88543800
H	4.41983500	2.99993200	-0.32772100
H	3.07244000	4.44073500	-1.84233500
C	1.16546000	-0.29370900	1.99677500
O	0.30698700	-0.91546100	2.66502700
O	2.41709200	-0.21970600	2.36433300
C	2.81725300	-0.98828800	3.51593600
H	3.88581900	-0.80848100	3.61968200
H	2.28384500	-0.64152900	4.40284600
H	2.62177400	-2.04689300	3.33856000
C	0.71330200	0.35465000	0.75813300
C	-3.94117000	-0.80843200	-0.97545300
C	-3.06066100	-0.13403000	-0.08648700
C	-5.28650300	-0.52359200	-0.98681000
C	-1.64429300	-0.36710500	-0.04195600
C	-3.59352900	0.89553700	0.74894100
C	-5.81560800	0.44889600	-0.11638800
H	-5.94073200	-1.05124200	-1.67266800
C	-0.81183700	0.36972800	0.86459900
C	-2.73897100	1.70456200	1.56966900
C	-4.98241500	1.14988700	0.72557500
H	-6.88089100	0.65763900	-0.12431000
C	-1.40698000	1.48995900	1.58632700

H	-0.74120200	-0.47852000	1.82012900
H	-3.18406800	2.50535200	2.15136200
H	-5.37950400	1.92087200	1.37889800
H	-0.74593000	2.10317600	2.19139200
H	-3.57135600	-1.54510800	-1.67216600
Cu	1.42391500	-0.81802000	-0.76689100
Cl	1.92025200	-2.81579600	0.18762900
Cl	1.86946800	-0.77797200	-2.96570800
O	-0.99343900	-1.23572800	-0.78882000
C	-1.45830500	-2.53905700	-1.20436800
H	-1.83659300	-2.48314100	-2.22521300
H	-0.56916700	-3.16847700	-1.17283000
H	-2.20793300	-2.91657500	-0.50802100

Structure and coordinates of 4-Int-p4.log

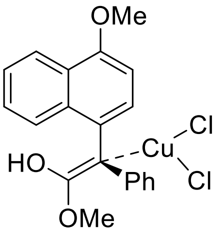

C	1.38485500	3.14838500	-1.39966600
C	0.71407100	2.07618300	-0.82785100
C	1.21063300	1.42892900	0.33319100
C	2.42585700	1.91266600	0.88530600
C	3.08335000	2.98673100	0.30342900
C	2.57087100	3.61027700	-0.83731900
H	0.97955300	3.61458800	-2.29163500
H	-0.21024300	1.73058700	-1.27722500
H	2.84481700	1.45368800	1.76980200
H	4.00713900	3.34429900	0.74717800
H	3.09695600	4.44860500	-1.28289300
C	0.42985700	0.27205300	0.84783500
C	0.82173700	-0.51569500	1.95746500
O	0.01291400	-1.38516800	2.51220700
O	2.00167400	-0.44176700	2.48823100
C	2.40036200	-1.39894500	3.49863100
H	3.44433900	-1.16736900	3.69753100
H	1.79696600	-1.26249800	4.39698100
H	2.30050700	-2.40984700	3.10300800
Cu	1.76311300	-0.48831600	-0.69644900
Cl	2.81773200	-0.15628700	-2.63374200
Cl	2.28063200	-2.59238900	-0.01400400
C	-3.11989700	1.59292900	0.95916800

C	-3.84533700	0.69565600	0.20634400
C	-3.20374200	-0.45058900	-0.36625600
C	-1.80573300	-0.65304100	-0.16139700
C	-1.06184600	0.31286800	0.60019700
C	-1.73832600	1.38726800	1.13971600
H	-5.00570300	-1.20891000	-1.28611700
C	-3.94653600	-1.38336800	-1.13472400
C	-1.22105600	-1.82033100	-0.72347000
H	-1.18383400	2.12449000	1.71408800
C	-1.96243500	-2.70970300	-1.46370100
C	-3.33967700	-2.48881700	-1.67978200
H	-0.17280200	-2.03720900	-0.54936200
H	-1.48236600	-3.59012900	-1.88009700
H	-3.91790700	-3.19381400	-2.26891600
O	-5.16386700	0.80203300	-0.04712500
H	-0.87261300	-1.34606700	2.10863200
H	-3.58341300	2.46286300	1.40798700
C	-5.87062400	1.91957600	0.47590500
H	-5.46760900	2.85861600	0.08102400
H	-6.90148400	1.79873700	0.14316100
H	-5.83882700	1.92906200	1.57092300

Structure and coordinates of 4-Int-o4.log

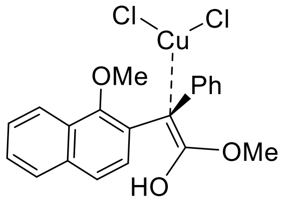

C	1.85060600	3.84853600	0.64464300
C	1.30166100	2.62453300	1.01688800
C	1.57383400	1.47225200	0.27323400
C	2.42233600	1.55626600	-0.83166700
C	2.97192400	2.78094800	-1.20520600
C	2.68486500	3.93097100	-0.47119800
H	1.62468400	4.73949400	1.22328600
H	0.64151600	2.56452700	1.87823300
H	2.65286100	0.65866700	-1.39757300
H	3.62601700	2.83369600	-2.07073500
H	3.11149600	4.88580300	-0.76410000
C	0.98150600	0.16276800	0.72306800
C	1.90979700	-0.68231600	1.38179100
Cu	0.47501200	-1.36287100	-0.73693400
Cl	2.47517300	-2.12521700	-1.43533500
Cl	-1.16601400	-2.59196100	-1.62981600

C	-0.48794700	0.03002300	0.86866000
C	-1.36516400	0.79940300	0.06617000
C	-2.78233800	0.68743200	0.19649600
C	-3.33194100	-0.21192900	1.14669700
C	-2.44527500	-0.95516000	1.97612300
C	-1.09577200	-0.83470200	1.85114100
H	-3.24129400	2.17331200	-1.31714900
C	-3.65703600	1.46908700	-0.60526400
C	-4.73830100	-0.32430000	1.26101900
H	-2.86000200	-1.62103100	2.72708400
H	-0.46381500	-1.40289200	2.51660100
C	-5.56571600	0.43244100	0.46342600
C	-5.01933200	1.33908100	-0.47413100
H	-5.15061800	-1.01661800	1.98965000
H	-6.64341900	0.33891200	0.55634200
H	-5.68117200	1.93809000	-1.09188300
O	1.54575400	-1.81238100	1.94626200
H	2.28709600	-2.38467900	2.20347400
O	3.15810900	-0.31111500	1.40303300
C	4.18956400	-1.18532400	1.90115600
H	5.11801000	-0.64203600	1.74091700
H	4.05102200	-1.36343500	2.97115100
H	4.21208000	-2.11615000	1.32789500
O	-0.91118500	1.73322300	-0.80652800
C	-0.69263100	1.31878000	-2.15751900
H	0.19589500	0.67857900	-2.22795800
H	-1.56393500	0.78888500	-2.55438500
H	-0.51672800	2.23162800	-2.72747700

Structure and coordinates of 4-Int-p5*.log

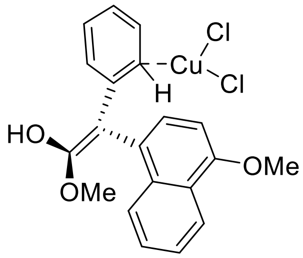

C	-1.13593900	-1.44489900	2.71226400
C	-0.24781300	-0.63206600	2.03174700
C	-0.68011200	0.51446500	1.30266800
C	-2.09097300	0.82027900	1.34150900
C	-2.97473800	-0.00519400	2.05320900
C	-2.50321400	-1.15487500	2.71340500
H	-0.76580600	-2.31585800	3.24248400

H	0.80800300	-0.87635700	2.04797900
H	-2.45301500	1.76584700	0.96657000
H	-4.01357400	0.29087000	2.16498900
H	-3.20125400	-1.79857700	3.23783000
C	0.27886000	1.21498400	0.51734800
C	-0.03319000	2.36247800	-0.25437000
O	0.81277400	2.71056600	-1.19169500
O	-1.09178500	3.07351800	-0.00959800
C	-1.55002500	4.08440700	-0.93346100
H	-2.51743200	4.39784200	-0.54750400
H	-0.86357100	4.93534300	-0.93090000
H	-1.67230600	3.64628000	-1.92715800
Cu	-2.97552200	-0.47562200	-0.12039700
Cl	-4.33714300	-2.24875600	-0.45624100
Cl	-2.27051600	0.61397600	-1.99748700
C	4.03385500	0.91885000	0.98820400
C	4.34822400	-0.20588400	0.25812000
C	3.32587700	-0.91830100	-0.45277100
C	1.98417700	-0.45004800	-0.39400000
C	1.67814800	0.71112500	0.39020000
C	2.69387300	1.36499900	1.04447000
H	4.65473300	-2.42688700	-1.25198700
C	3.62922100	-2.07636900	-1.21474100
C	0.98911100	-1.15531700	-1.12264500
H	2.46687200	2.24900600	1.63474200
C	1.30794100	-2.27394800	-1.85407800
C	2.64015000	-2.74369800	-1.89768400
H	-0.03422700	-0.79234200	-1.11364900
H	0.53084300	-2.79575300	-2.40467900
H	2.88209900	-3.63023900	-2.47619200
O	5.59015000	-0.72234100	0.14569200
H	4.79217700	1.47019700	1.53074400
C	6.65987400	-0.06477900	0.81055300
H	6.49949300	-0.04718800	1.89433300
H	7.55323800	-0.64763700	0.58524300
H	6.78818700	0.95699700	0.43607600
H	0.55691800	3.51157300	-1.67889600

Structure and coordinates of 4-Int-o5*.log

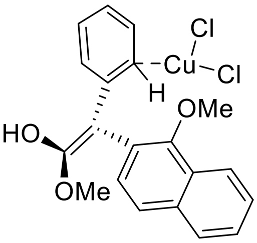

C	3.06798200	-0.79788400	-1.81486000
C	2.14045900	0.20090900	-1.47640400
C	0.75112800	-0.13673700	-1.27319000
C	0.40085500	-1.51757300	-1.38691800
C	1.33374200	-2.48904200	-1.69171400
C	2.67306400	-2.14406400	-1.90393700
H	4.08187600	-0.51571800	-2.08353700
H	2.44016300	1.23461200	-1.55904500
H	-0.62660100	-1.81519700	-1.21428500
H	1.02539800	-3.52698900	-1.75735700
H	3.40758000	-2.90619900	-2.13960900
C	-4.28566900	-0.43797100	1.76614100
C	-3.64645400	-0.27117400	0.50854200
C	-5.59293500	-0.85568400	1.83004700
C	-2.28847100	0.15622500	0.40554000
C	-4.37424500	-0.53443100	-0.68588000
C	-6.31937500	-1.12478300	0.64371200
H	-6.07653600	-0.97948400	2.79432900
C	-1.69043300	0.33154600	-0.82358700
C	-3.73645000	-0.35206600	-1.94307200
C	-5.72517600	-0.96464600	-0.58398800
H	-7.35213800	-1.45377000	0.71047900
C	-2.43520200	0.07106700	-2.00661600
H	-4.29408800	-0.55048100	-2.85401600
H	-6.27951300	-1.16327000	-1.49747100
H	-1.95080500	0.21093100	-2.96880500
H	-3.73013700	-0.22264600	2.67309600
Cu	3.12452400	-0.37701700	0.35596900
Cl	4.40890200	-1.99290700	1.30690100
Cl	2.47905900	1.27790700	1.76049300
C	-0.27370700	0.78929100	-0.93192600
C	-0.05366100	2.16004400	-0.66329600
O	-1.07410000	2.86393700	-0.23590800
O	1.10283600	2.71361600	-0.86437600
C	1.39882400	4.04762600	-0.40163800
H	2.46371800	4.17515100	-0.58101100
H	0.83909700	4.78308900	-0.98679200
H	1.19741700	4.12762100	0.66911900

H	-0.87140600	3.80143200	-0.08199200
O	-1.59679900	0.43280000	1.55168200
C	-0.89486400	-0.68387000	2.10397300
H	-0.10899800	-1.02839700	1.42339700
H	-0.43972900	-0.33577400	3.03123100
H	-1.58381600	-1.50994100	2.31670500
